# Real-Case Validation of a Weather-Driven Two-Stage Geese V-Formation Algorithm for Distributed Generation Planning and Voltage-Security Assessment in Multi-Feeder Distribution Networks

**DOI:** 10.3390/s26165086

**Published:** 2026-08-11

**Authors:** Omar Yaseen Saeed, Carlos Roldán-Blay, Carlos Roldán-Porta

**Affiliations:** 1 Institute for Energy Engineering, Universitat Politècnica de València, Camino de Vera, s/n, Edificio 8E, Escalera F, 5^a^ Planta, 46022 Valencia, Spain; carrolbl@die.upv.es (C.R.-B.); croldan@die.upv.es (C.R.-P.); 2 Scientific Research Commission, Baghdad 10071, Iraq

**Keywords:** active distribution networks, battery energy storage systems, distributed energy resources, Geese V-Formation Algorithm, inter-feeder energy exchange, optimal placement and sizing, weather-driven optimization

## Abstract

High penetration of distributed energy resources (DERs) is reshaping radial distribution networks, yet weather-dependent generation, variable demand, and feeder-level surplus–deficit imbalance can compromise voltage quality and coordinated operation. Existing planning approaches often optimize feeders independently and therefore provide limited insight into how local DER portfolios should support inter-feeder energy exchange under time-varying conditions. This study proposes a weather-driven two-stage Geese V-Formation Algorithm (GVFA) framework for planning DER integration and feeder coordination in a practical five-feeder 11 kV Tajeeyaat/North Baghdad system, with complementary validation on a five-instance IEEE 33-bus benchmark cluster. Stage 1 optimizes the siting and sizing of photovoltaic units, wind turbines, battery energy storage systems, capacitor banks, and feeder-specific auxiliary resources using backward/forward-sweep load flow. Stage 2 uses hourly surplus–deficit profiles to select tie-switch configurations and exchange capacities for feeder-to-feeder energy sharing. The framework is evaluated through convergence analysis, optimizer comparison, N-1 contingencies, and seasonal load-growth tests. For the practical system, 24 h aggregate losses decreased from 11,258.1571 to 3367.8481 kWh-eq, corresponding to a 70.0853% reduction. The minimum-voltage range improved from 0.9497–0.9898 to 0.9897–0.9997 p.u., while grid-import reduction reached 94.0270%. For the IEEE-33 cluster, 24 h aggregate losses decreased from 31,746.4712 to 6381.1403 kWh-eq, corresponding to a 79.8997% reduction. The minimum-voltage range improved from 0.8268–0.8632 to 0.9465–0.9683 p.u., while grid-import reduction reached 84.4596%. The framework provides a planning-oriented, sensor-ready decision-support basis for DER siting, voltage-support assessment, grid-import reduction, and candidate inter-feeder exchange corridors.

## 1. Introduction

The transition from passive radial feeders to active distribution networks is being accelerated by the increasing penetration of photovoltaic (PV) generation, wind turbines (WTs), battery energy storage systems (BESSs), electric vehicles (EVs), flexible demand, and dispatchable distributed generation. These technologies are selected in this study because they represent complementary flexibility mechanisms in modern distribution-network planning. PV generation is widely deployable at residential, commercial, and utility scales and is directly linked to local solar-resource availability. WTs provide an additional renewable source whose temporal behavior differs from PV generation and can therefore diversify feeder-level renewable output. BESS units provide temporal flexibility by absorbing surplus renewable energy and supporting feeders during high-demand periods. EV charging stations represent an emerging source of demand growth and, when coordinated with PV and storage, may also provide flexibility through charging management or bidirectional energy exchange. Capacitor banks remain important for reactive-power compensation and voltage-profile support in radial feeders with high (R/X) ratios. Feeder-specific auxiliary resources, such as diesel or biogas generation, provide dispatchable support during renewable-generation shortages or stressed operating conditions.

Although these resources can reduce upstream energy procurement and defer conventional network reinforcement, they also alter branch-current patterns, create bidirectional power flows, increase voltage variability, and complicate feeder operation under uncertain renewable-generation and demand conditions. The diversity of DER modeling assumptions, simulation approaches, and performance metrics remains a major barrier to objective comparison among planning studies [1]. Moreover, distributed generation can improve efficiency and reliability only when placement, sizing, and control are coordinated; otherwise, voltage deviation, reverse power flow, protection miscoordination, and power-quality concerns may become more severe [2].

Long-term distribution expansion planning has demonstrated the value of renewable distributed generation in reducing network losses and postponing investment in conventional assets [3]. However, the practical value of such planning decisions depends on whether the selected DER portfolios remain technically feasible under hourly and seasonal variations in solar irradiance, wind speed, EV-related demand, and load growth. Recent reviews of high-DER active distribution networks therefore call for optimization frameworks that integrate planning and representative operating conditions rather than treating DER capacities as fixed deterministic inputs [4]. This requirement is particularly important in radial feeders, where electrical distance from the source, branch impedance, and local reactive-power balance strongly influence the relationship between DER injection, voltage recovery, and loss reduction.

Uncertainty-aware allocation studies have improved the representation of PV, WT, and load variability. Monte Carlo scenarios, backward reduction, and metaheuristic optimization have been combined to determine renewable DG siting and sizing under time-varying load and renewable uncertainty [5]. These results confirm that uncertainty should be included during allocation rather than considered only as a post-optimization sensitivity test. Correlations among renewable-generation variables have also been modeled during simultaneous DG and capacitor-bank allocation [6]. This is important because correlated weather conditions influence both active generation and reactive-power requirements. However, such formulations remain primarily feeder-level allocation models and do not address how adjacent feeders can share surplus renewable energy after local resources have been optimally installed. Similarly, PV–WT allocation under uncertainty has been studied using sensitivity-based candidate selection and metaheuristic search [7], but such formulations do not fully exploit the temporal flexibility offered by BESSs or the planning role of inter-feeder exchange.

PV–storage allocation models have evolved from static capacity planning toward security-aware and economic formulations. A bi-level PV energy-storage allocation approach with a day-ahead dispatch layer and a security-region concept has strengthened the connection between investment decisions and operating feasibility [8]. Joint DG and BESS planning has further shown that storage can improve loss reduction and reliability beyond what is achievable by DG alone [9]. Nevertheless, the economic and reliability benefits of BESSs are sensitive to aging, ambient temperature, replacement requirements, and charging/discharging constraints [10]. Therefore, planning studies must clearly distinguish between using BESSs as a planning-level flexibility resource and claiming detailed chronological battery operation.

Voltage security in DER-rich feeders cannot be assured by active-power placement alone. Coordinated Volt/VAR control and energy-storage scheduling can mitigate PV-driven voltage deviations under forecast uncertainty [11]. This highlights the need to couple active-power support with reactive-power compensation. However, many related studies focus on mobile storage scheduling or operational voltage control rather than long-term allocation of multiple stationary DERs and capacitor banks. PV, EV demand, BESS installation, replacement, and maintenance costs have also been incorporated using metaheuristic algorithms [12]. Although this provides an important techno-economic perspective, coordination of reactive compensation devices and feeder collaboration remains limited.

Recent PV–EV studies further justify the inclusion of EV-related flexibility in active distribution-network planning. A predictive optimization strategy for solar PV–EV systems with vehicle-to-grid (V2G) and vehicle-to-home (V2H) modes has shown that coordinated PV and EV energy management can reduce grid stress and daily electricity cost under forecast-informed operation [13]. However, such work is primarily focused on household or local energy-management operation. It does not address multi-feeder DER siting, capacitor-bank allocation, radial load-flow constraints, or planning-level inter-feeder exchange corridors. This distinction motivates the inclusion of EVCS-related auxiliary resources in the present framework while keeping the main contribution focused on distribution-feeder planning rather than household energy-management control.

Recent work on energy-storage operation has sought to combine economic objectives with network constraints. Salp swarm optimization has been applied to storage operation in active distribution networks [14], while source–load uncertainty, branch constraints, and BESS operating behavior have been considered in related formulations [15]. However, critical reviews of simultaneous DG–capacitor placement indicate that many published methods still rely on simplified loading assumptions, limited uncertainty treatment, or isolated technical objectives [16]. Consequently, a coordinated model should distinguish between active-power balancing by PV, WT, BESS, and auxiliary generation, and reactive-power compensation by capacitor banks and inverter capability.

Distribution-network reconfiguration is a well-established approach for changing power-flow paths, reducing losses, and improving voltage profiles. Simultaneous DG placement and network reconfiguration have been formulated as mixed-integer linear programming problems, providing rigorous benchmarks for radiality-preserving topology optimization [17]. However, deterministic MILP formulations may become computationally restrictive when multiple stochastic DER profiles, nonlinear operating constraints, and continuous sizing variables are added. Modern reconfiguration research also needs to distinguish between static planning and dynamic switching, since the latter requires communication infrastructure, switch availability, protection compatibility, and operational feasibility [18].

Two-stage models provide a promising mechanism for separating long-term asset-allocation decisions from short-term or higher-level operational adaptation. Robust two-stage optimization has been developed for source–load uncertainty, where first-stage resource decisions are adjusted by second-stage operational variables [19]. Multi-scenario collaborative optimization incorporating EVs and mobile energy storage has also shown that spatial–temporal flexibility can improve PV accommodation and address voltage violations [20]. Nevertheless, these formulations are not specifically designed to use optimized feeder boundary profiles as inputs to a second-stage feeder-to-feeder exchange-planning layer. This distinction is important because practical utilities often operate groups of adjacent radial feeders whose local DER surplus and deficit conditions vary hourly.

More recent research has combined storage and reconfiguration using increasingly sophisticated multi-objective formulations. Mobile and stationary storage have been optimized to reduce reverse power flow, peak demand, ramp rate, and losses under high renewable penetration [21]. Mobile energy storage and active distribution-network operation have also been coordinated through Stackelberg game frameworks [22]. Additional studies have addressed active–reactive coordination with mobile storage and dynamic reconfiguration [23], while mobile BESSs and dynamic feeder reconfiguration have been combined using NSGA-III to improve voltage indices, losses, operating cost, and emissions [24]. In addition, a White Shark Optimizer-based energy-management strategy has been developed for a PV/PEMFC/lithium–ion battery/supercapacitor hybrid microgrid to allocate power among sources while minimizing hydrogen consumption under operational constraints [25]. Compared with state-machine control, conventional EEMS, and PSO-based EEMS, that strategy reduced hydrogen use by 34.17%, 29.47%, and 2.10%, respectively, while improving overall system efficiency.

### 1.1. Research Gaps

The reviewed literature reveals five unresolved issues. First, many DER-allocation studies remain feeder-local and therefore cannot quantify how optimized local surpluses can support neighboring feeders through controllable tie connections. Second, uncertainty is often represented through probabilistic scenarios or robust sets, but its integration with weather-driven hourly DER profiles, capacitor-bank reactive support, and feeder exchange remains limited. Third, BESS studies frequently emphasize cost, placement, or dispatch separately; relatively few planning frameworks jointly optimize multiple BESS units with PV, WT, capacitor banks, and auxiliary DERs under network-voltage constraints while clearly distinguishing planning-level storage representation from detailed chronological battery dispatch. Fourth, reconfiguration studies often optimize internal feeder topology, whereas planning inter-feeder tie-switch capacity for energy exchange among already optimized feeder portfolios has received less attention. Fifth, many advanced metaheuristic studies report promising numerical performance, but comparisons are often performed under different objectives, stopping criteria, decision dimensions, or test conditions. Accordingly, algorithmic superiority should not be inferred from normalized convergence curves alone without consistent computational settings.

### 1.2. Role and Contribution of the Present Study

The present research addresses these gaps through a weather-driven, hierarchical, and parallelizable two-stage GVFA framework implemented in Python. Stage 1 performs feeder-level allocation and sizing of multiple PV units, WT generation, BESS units, capacitor banks, and feeder-specific auxiliary resources, including biogas, diesel generation, and EVCS-related flexibility, using radial backward/forward-sweep load flow. The objective formulation evaluates active-loss reduction, voltage deviation, voltage-limit penalties, grid import, placement suitability, and bounded export potential. This structure explicitly links DER decisions to both active- and reactive-power-flow mechanisms.

Stage 2 uses the optimized feeder-level surplus and deficit profiles obtained from Stage 1 to determine tie-switch states and exchange capacities among feeders. Hence, the proposed model does not treat feeder exchange as an assumed benefit; it evaluates whether local energy transfer is technically advantageous after considering losses, connectivity, import dependence, and exchange-capacity constraints. This is a substantive distinction from isolated DER-siting studies and from conventional internal feeder-reconfiguration models.

The proposed GVFA framework builds upon the earlier collaborative interconnected-microgrid planning study [26], which demonstrated the applicability of GVFA under intermittent DERs and dynamic loads. However, the present work does not claim GVFA itself as a new algorithm. Instead, its novelty lies in reformulating GVFA within a hierarchical feeder-planning structure that combines local DER/CAP siting and sizing with a second-stage inter-feeder exchange layer. The practical Tajeeyaat/North Baghdad feeder cluster is therefore used as a real utility validation case, not as the main scientific contribution by itself. The main scientific contribution is the weather-driven, two-stage, multi-feeder planning formulation and its validation under benchmark and real utility-derived network conditions.

The principal contributions of this work are summarized as follows:

- A weather-driven, hierarchical, and parallelizable two-stage GVFA planning framework is developed for active radial distribution networks with high DER penetration.
- A feeder-level Stage 1 formulation is proposed to optimize the siting and sizing of PV units, WTs, BESSs, capacitor banks, and feeder-specific auxiliary resources under radial load-flow and voltage-security constraints.
- A Stage 2 inter-feeder coordination layer is introduced to use weather-dependent feeder surplus–deficit profiles for selecting candidate tie-switch states and exchange capacities.
- The framework explicitly links active-power resources, reactive-power compensation, grid-import reduction, bounded export potential, and feeder-to-feeder exchange within a unified planning workflow.
- The proposed method is validated on both a five-instance IEEE 33-bus benchmark cluster and a practical five-feeder 11 kV Tajeeyaat/North Baghdad distribution system reconstructed from utility-derived network data.
- Robustness is assessed through convergence analysis, N-1 contingency evaluation, seasonal load-growth sensitivity, post-optimal measurement-input uncertainty assessment, and optimizer comparison under consistent computational settings.

The remainder of this paper is organized as follows. Section 2 presents the proposed weather-driven two-stage GVFA framework, including the radial distribution-network model, PV, WT, BESSs, capacitor-bank, and auxiliary-resource representations; the backward/forward-sweep load-flow procedure; the Stage 1 DER placement and sizing formulation; and the Stage 2 inter-feeder energy-exchange optimization. Section 3 reports and critically discusses the results obtained for the IEEE 33-bus benchmark cluster and the practical Tajeeyaat/North Baghdad 11 kV feeder system, with emphasis on loss reduction, voltage-profile enhancement, energy exchange, convergence, N-1 contingency performance, seasonal load-growth sensitivity, measurement-input uncertainty, and optimizer comparison. Finally, Section 4 summarizes the main findings, outlines the practical implications of the proposed framework for active distribution-network planning, and suggests directions for future research.

## 2. Methodology

### 2.1. Overall Framework of the Proposed Method

The proposed method is a Python-implemented, weather-driven, two-stage planning framework for optimal allocation of distributed energy resources (DERs) in radial active distribution networks. It is designed to preserve the electrical realism required in distribution-system studies while keeping the search problem tractable for multi-resource and multi-feeder planning. Two test environments are considered: a benchmark cluster composed of five IEEE 33-bus feeder instances and a real five-feeder 11 kV Tajeeyaat/North Baghdad system based on Baghdad Electricity Distribution Company data. The two cases share the same modeling philosophy, backward/forward sweep (BFS) power-flow engine, resource models, Geese V-Formation Algorithm (GVFA), constraint treatment, and post-processing workflow. Their distinction lies in the source of the network data: the IEEE-33 system is embedded in the code, whereas the practical system is reconstructed from load, overhead-line, and node records in an Excel workbook.

**Figure 1:**
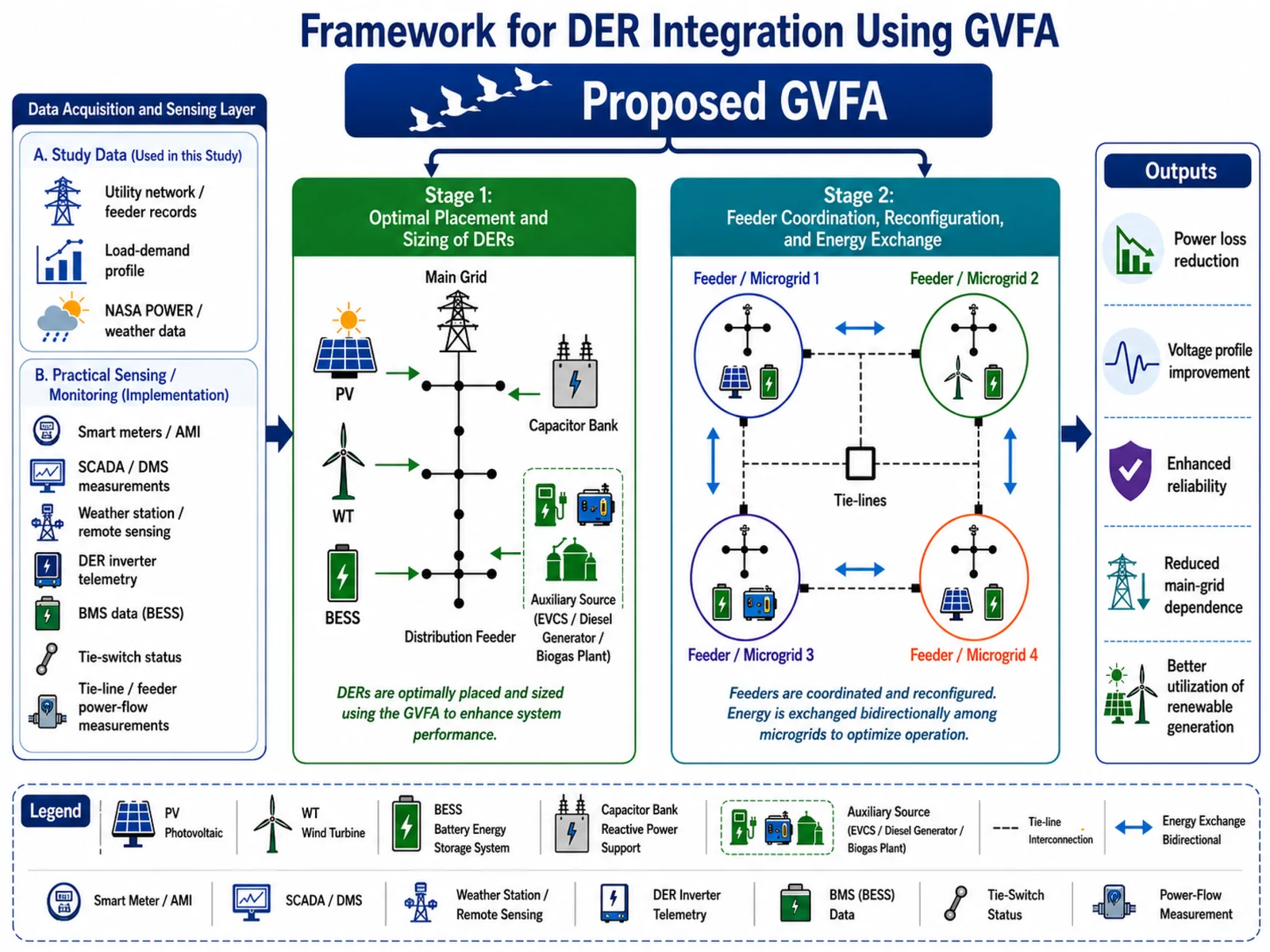
Conceptual architecture of the proposed GVFA-based DER integration framework. Stage 1 determines the feeder-level placement and sizing of PV, WT, BESSs, capacitor banks, and an auxiliary resource; Stage 2 coordinates selected feeder ties and bidirectional energy exchange.

Figure 1 provides the high-level physical and functional interpretation of the proposed architecture. The left-hand input block represents feeder topology, demand profile, and weather variables that parameterize the planning problem. In Stage 1, the schematic distinguishes active-power resources, namely PV, WT, BESSs, and the auxiliary unit, from capacitor-bank reactive support. All resources are connected to the same radial feeder and assessed through the BFS model. Stage 2 is represented as a coordination layer above individual feeder solutions. Its tie-lines represent candidate feeder-level interconnections through which feasible surplus–deficit transfers are scheduled. Therefore, the interconnections in Figure 1 are conceptual representations of the exchange graph; their final switch states and capacities are decision-dependent outputs of the Stage 2 formulation rather than fixed physical assumptions. The output block summarizes the technical performance indicators addressed by the implemented objectives, namely power-loss reduction, voltage-profile improvement, enhanced supply support, reduced main-grid dependence, and better utilization of locally available renewable generation.

The optimization is divided into two coordinated stages. Stage 1 optimizes each feeder independently. A GVFA candidate determines the locations and sizes of four photovoltaic (PV) units, one wind turbine (WT), four battery energy storage system (BESS) units, two capacitor banks (CAPs), and one auxiliary resource. The auxiliary resource is selected deterministically as an electric-vehicle charging station (EVCS), diesel generator, or biogas generator, depending on the feeder configuration. Each device is represented by a normalized location gene and a normalized size gene. The former is mapped to a feasible candidate bus, whereas the latter is translated into a technology-specific capacity. The decoded portfolio is assessed through weather-scaled hourly injections, BFS load flow, loss calculation, voltage assessment, penalty evaluation, and export-potential evaluation.

Stage 2 operates on the optimized net-import and net-export profiles produced by Stage 1. It identifies normally open feeder-to-feeder ties and their transfer capacities. Surplus energy from one feeder is transferred to an importing feeder only when a selected tie exists and its capacity is sufficient. This separation between local resource planning and inter-feeder coordination is deliberate. It prevents the Stage 2 solution from masking an inadequate Stage 1 portfolio, while allowing local flexibility to be exploited at the cluster level. The final outputs include DER placement tables, capacitor-bank summaries, feeder loss and voltage indices, tie-switch configurations, hourly and seasonal exchange results, N-1 contingency results, load-growth sensitivity results, GVFA parameter-sensitivity results, convergence curves, and publication-oriented tables and figures.

### 2.2. Base-Case System Design and Input Data Configuration

Table 1 clarifies the base-case modeling boundary before presenting optimization results, which is essential because the IEEE-33 cluster and the practical Tajeeyaat/North Baghdad system use different data origins but share the same two-stage GVFA workflow. It also makes transparent that PV, WT, BESSs, capacitor banks, EVCSs, DG, and biogas were selected to represent complementary active-power, reactive-power, storage, electrification, and dispatchable-support mechanisms. The most important technical caveat is that EVCSs and biogas are treated as predefined auxiliary planning scenarios rather than utility-measured installed assets. This distinction strengthens the manuscript by preventing overinterpretation of planning assumptions as field-validated deployment data.  

### 2.3. Sensing, Measurement-Data Layer, and Practical Data Requirements

The proposed framework is developed as an offline planning model. Therefore, it is necessary to distinguish clearly between the data actually used in this study and the sensing or monitoring infrastructure required for practical implementation. In the present study, the benchmark system is represented using embedded IEEE 33-bus data, while the practical Tajeeyaat/North Baghdad system is represented using utility-derived feeder records, including topology, line data, load information, and node records. Weather-dependent renewable-resource modeling is based on NASA POWER weather data for Baghdad/North Baghdad.

For practical deployment, the same planning framework would require a synchronized measurement layer capable of updating demand, voltage, power flow, DER output availability, battery status, capacitor status, tie-switch position, and feeder-exchange conditions. Smart meters and advanced metering infrastructure (AMI) would provide demand profiles; SCADA/DMS would provide voltage, current, power-flow, and switch-status measurements; weather stations or remote-sensing data would provide irradiance and wind information; DER inverter telemetry would provide PV/WT availability; BMS measurements would provide BESS status; and tie-line meters or switch-position sensors would verify feeder-exchange feasibility. Table 2 summarizes the relationship between each input variable, its source in the present offline study, its practical sensing counterpart, the expected time resolution, and its use in Stage 1 or Stage 2.

Accordingly, the present work should be interpreted as a planning-level assessment based on historical, benchmark, and utility-derived data. It does not claim that real-time AMI, SCADA, DER inverter telemetry, BMS, or tie-switch measurements were used in the present offline simulations. Rather, Table 2 identifies the measurement infrastructure that would be required for future practical implementation.

### 2.4. Distribution Network and Load-Flow Model

Each feeder is represented as a connected radial graph, as expressed in Equation (1):

(1) $$G_{f}=\left(B_{f},L_{f}\right),$$

where $B_{f}$ and $L_{f}$ are, respectively, the bus and branch sets of feeder *f*. A branch ℓ=(i,j) connects a parent bus *i* to a child bus *j* and has the series impedance defined in Equation (2):

(2) $$Z_{ij}=R_{ij}+jX_{ij}.$$

The standard per-unit base impedance is calculated using Equation (3):

(3) $$Z_{\mathrm{base}}=\frac{V_{\mathrm{base}}^{2}}{S_{\mathrm{base}}}.$$

This expression follows standard power-system per-unit modeling practice. The IEEE-33 model uses $V_{\mathrm{base}}=12.66\;\mathrm{kV}$ and $S_{\mathrm{base}}=10\;\mathrm{MVA}$, whereas the practical feeders use $V_{\mathrm{base}}=11\;\mathrm{kV}$ and the same apparent-power base. This common base permits consistent numerical scaling without modifying the actual branch topology or load distribution.

At bus *i*, the nominal active and reactive demands are $P_{i}^{L}$ and $Q_{i}^{L}$. For month *m* and hour *h*, a weather- and season-dependent multiplier $\lambda_{m,h}$ modifies the demand. The net active and reactive powers used in the load flow are defined in Equations (4) and (5):

(4) $$\begin{array}{cc} P_{i,m,h}^{\mathrm{net}} & =\lambda_{m,h}P_{i}^{L}-P_{i,m,h}^{\mathrm{inj}}, \end{array}$$

(5) $$\begin{array}{cc} Q_{i,m,h}^{\mathrm{net}} & =\lambda_{m,h}Q_{i}^{L}-Q_{i,m,h}^{\mathrm{inj}}. \end{array}$$

Here, $P_{i,m,h}^{\mathrm{inj}}$ and $Q_{i,m,h}^{\mathrm{inj}}$ aggregate the contributions of PV, WT, BESSs, CAP, and auxiliary devices at the bus. This sign convention correctly represents DER generation as negative net demand while retaining a single radial load-flow formulation.

The BFS method is adopted because the studied systems are radial and exhibit the relatively high R/X ratios typical of medium-voltage feeders. The current-injection, backward-sweep, forward-sweep, and loss equations are standard distribution-load-flow relations and should be read together with classical distribution power-flow and reconfiguration references. At iteration *k*, the complex current injection is calculated using Equation (6):

(6) $$I_{i,m,h}^{\left(k\right)}={\left(\frac{S_{i,m,h}^{\mathrm{net}}}{V_{i,m,h}^{\left(k\right)}}\right)}^{*},$$

where ${\left(\;\cdot \;\right)}^{*}$ denotes complex conjugation. During the backward sweep, downstream current contributions are accumulated toward the root bus. During the forward sweep, the voltage at child bus *j* is updated using Equation (7):

(7) $${\tilde{V}}_{j,m,h}^{\left(k+1\right)}=V_{i,m,h}^{\left(k+1\right)}-Z_{ij}^{\mathrm{pu}}I_{ij,m,h}^{\left(k\right)}.$$

To improve numerical robustness under high DER injections, the code applies voltage damping, as shown in Equation (8):

(8) $$V_{j,m,h}^{\left(k+1\right)}=\alpha_{\mathrm{BFS}}{\tilde{V}}_{j,m,h}^{\left(k+1\right)}+\left(1-\alpha_{\mathrm{BFS}}\right)V_{j,m,h}^{\left(k\right)}.$$

The damping factor is set to $\alpha_{\mathrm{BFS}}=0.60$. This is a numerical implementation parameter used to stabilize BFS convergence under high DER penetration and possible reverse-power-flow conditions. It is not an electrical device parameter.

Convergence is declared using Equation (9):

(9) $$\max_{i\in B_{f}}\left|V_{i,m,h}^{\left(k+1\right)}-V_{i,m,h}^{\left(k\right)}\right|<10^{-7},$$

or after 100 iterations. The tolerance $10^{-7}$ is adopted to obtain repeatable voltage and loss calculations without excessive computational burden, while the iteration cap of 100 prevents non-convergent candidates from consuming unlimited runtime.

The active and reactive branch-loss components are obtained from Equations (10) and (11):   

(10) $$\begin{array}{cc} P_{m,h}^{\mathrm{loss}}\; & =S_{\mathrm{base},\mathrm{kW}}\sum_{\ell \in L_{f}}{\left|I_{\ell ,m,h}\right|}^{2}R_{\ell }^{\mathrm{pu}}, \end{array}$$

(11) $$\begin{array}{cc} Q_{m,h}^{\mathrm{loss}}\; & =S_{\mathrm{base},\mathrm{kVAr}}\sum_{\ell \in L_{f}}{\left|I_{\ell ,m,h}\right|}^{2}X_{\ell }^{\mathrm{pu}}. \end{array}$$

These expressions are standard $I^{2}R$ and $I^{2}X$ branch-loss relations expressed in per-unit form and converted back to kW and kVAr using the corresponding base quantities.

### 2.5. Weather-Driven DER and Capacitor Models

The PV model is intentionally planning-oriented. Rather than relying on a detailed semiconductor model, it combines the optimized PV rating $S_{r}^{\mathrm{PV}}$, a normalized hourly profile $\phi_{h}^{\mathrm{PV}}$, and a monthly irradiance factor $\gamma_{m}^{\mathrm{PV}}$, as shown in Equation (12):

(12) $$P_{r,m,h}^{\mathrm{PV}}=S_{r}^{\mathrm{PV}}\phi_{h}^{\mathrm{PV}}\gamma_{m}^{\mathrm{PV}}.$$

The solar factor is generated from the monthly all-sky shortwave irradiation $G_{m}$ using Equation (13):

(13) $$\gamma_{m}^{\mathrm{PV}}=\mathrm{clip}\left(\frac{G_{m}}{5.0},0.35,1.22\right).$$

The denominator $5.0\;\mathrm{kWh}/m^{2}/\mathrm{day}$ is a representative planning reference for monthly average daily shortwave irradiation. It converts $G_{m}$ into a dimensionless seasonal multiplier and is not a PV efficiency or a rated irradiance value. The lower clipping value of 0.35 avoids unrealistically low PV availability in low-irradiance months, while the upper clipping value of 1.22 prevents excessive amplification in high-irradiance months. These constants are empirical planning saturation limits used to preserve seasonal variation while maintaining numerical realism.

PV reactive support is represented by the fixed ratio in Equation (14):

(14) $$Q_{r,m,h}^{\mathrm{PV}}=0.18\left|P_{r,m,h}^{\mathrm{PV}}\right|.$$

The coefficient 0.18 represents a planning-level inverter reactive-support contribution. It is used to reflect the voltage-support capability of inverter-interfaced PV without modeling a full inverter capability curve, grid-code control law, or real-time Volt/VAr controller.

The PV rating bound is given in Equation (15):

(15) $$0\leq S_{r}^{\mathrm{PV}}\leq \min\left(2600,\;1.05P_{f}^{\mathrm{peak}}\right)\;\left[\mathrm{kW}\right].$$

The value 2600 kW is the individual PV capacity cap used in the final simulations. The factor $1.05P_{f}^{\mathrm{peak}}$ prevents a single PV unit from becoming disproportionately large relative to the feeder peak demand.

WT generation is represented by a normalized, weather-scaled profile. The active output is calculated using Equation (16):

(16) $$P_{m,h}^{\mathrm{WT}}=S^{\mathrm{WT}}\mathrm{clip}\left(\phi_{h}^{\mathrm{WT}}\gamma_{m}^{\mathrm{WT}}+\epsilon_{h},0.05,0.98\right),$$

where $\epsilon_{h}$ is a small seeded perturbation. The monthly wind factor is defined in Equation (17):

(17) $$\gamma_{m}^{\mathrm{WT}}=\mathrm{clip}\left[{\left(\frac{v_{m}}{4.0}\right)}^{3},0.20,1.18\right].$$

The denominator 4.0m/s is a representative wind-speed reference used to convert monthly wind speed into a dimensionless availability multiplier. The cubic relationship reflects the approximate dependence of wind power on wind speed. The clipping limits 0.20 and 1.18 prevent unrealistically low or excessive seasonal wind multipliers. Similarly, the output-profile clipping limits 0.05 and 0.98 prevent negative WT output and avoid assuming rated output in all hours. This is a planning approximation and should not be interpreted as an explicit manufacturer-specific cut-in, rated, and cut-out wind-turbine curve. WT reactive support uses a fixed ratio of 0.12, representing a lower planning-level reactive contribution than the inverter-based PV and BESS resources.

The BESS is treated as a planning-level flexible power resource rather than a detailed chronological battery-dispatch model. For unit *r*, Equation (18) defines the active and reactive BESS contributions:

(18) $$P_{r,m,h}^{\mathrm{BESS}}=S_{r}^{\mathrm{BESS}}\phi_{h}^{\mathrm{BESS}},\;Q_{r,m,h}^{\mathrm{BESS}}=0.25\left|P_{r,m,h}^{\mathrm{BESS}}\right|.$$

Positive and negative values of $\phi_{h}^{\mathrm{BESS}}$ represent discharge and charge, respectively. The coefficient 0.25 is a planning-level reactive-power support ratio associated with the inverter-coupled nature of the BESS. It is not a fixed operational power factor, and it does not represent a detailed converter capability curve. It allows the planning model to reflect the stronger voltage-support potential of inverter-coupled storage compared with WT units.

The BESS rating bound is given in Equation (19):

(19) $$0\leq S_{r}^{\mathrm{BESS}}\leq \min\left(2000,\;0.85P_{f}^{\mathrm{peak}}\right)\;\left[\mathrm{kW}\right].$$

The value 2000 kW is the individual BESS power cap used in the final simulations, while the factor $0.85P_{f}^{\mathrm{peak}}$ prevents a single BESS unit from dominating the feeder peak demand.

Accordingly, the BESS representation is planning-oriented and uses a prescribed normalized flexibility profile to quantify its contribution to DER siting, feeder losses, voltage support, grid-import reduction, and inter-feeder exchange potential. It does not model chronological state-of-charge trajectories, energy-capacity optimization, charging/discharging efficiency, cycle-by-cycle degradation, converter dynamics, market dispatch, replacement cost, or detailed energy-management operation. Therefore, BESS-related conclusions in this work should be interpreted as planning-level network-support effects, not as evidence of validated operational BESS dispatch.

Capacitor banks inject reactive power only, as expressed in Equation (20):

(20) $$P_{r,m,h}^{\mathrm{CAP}}=0,\;Q_{r,m,h}^{\mathrm{CAP}}=S_{r}^{\mathrm{CAP}}\phi_{m,h}^{\mathrm{CAP}}.$$

Their load-dependent availability is defined in Equation (21):

(21) $$\phi_{m,h}^{\mathrm{CAP}}=\mathrm{clip}\left[\phi_{\min}+\left(1-\phi_{\min}\right){\left(\frac{\lambda_{m,h}}{\max_{h}\lambda_{m,h}}\right)}^{\beta },0,1\right],$$

where $\phi_{\min}=0.30$ and β=0.85. The parameter $\phi_{\min}=0.30$ represents minimum reactive support availability, while β=0.85 controls the nonlinear increase in capacitor utilization with loading. The clipping limits 0 and 1 ensure that the capacitor availability factor remains physically interpretable. This formulation permits stronger reactive support at heavily loaded hours while avoiding physically implausible full compensation at light load.

The capacitor-bank rating constraint is expressed in Equation (22):

(22) $$0\leq S_{r}^{\mathrm{CAP}}\leq \min\left(1400,\;0.55P_{f}^{\mathrm{peak}}\right)\;\left[\mathrm{kVAr}\right].$$

The value 1400 kVAr limits the individual capacitor size, while $0.55P_{f}^{\mathrm{peak}}$ prevents excessive reactive-power compensation relative to feeder loading.

### 2.6. Two-Stage Optimization Formulation and Constraints

The mathematical partitioning follows the architecture shown in Figure 1 and Table 3. Stage 1 corresponds to the feeder-level resource-integration layer: it determines device locations and sizes while enforcing voltage feasibility through repeated BFS evaluations. Stage 2 corresponds to the coordination layer: it does not alter the internal radial branches of a feeder, but selects feeder-pair tie states and exchange capacities after the local portfolios have been determined. This separation preserves the causal order between local adequacy and inter-feeder assistance; energy exchange is used to coordinate feasible portfolios rather than to conceal an infeasible local placement decision.

For feeder *f*, the proposed Stage 1 GVFA objective is given in Equation (23):

(23) $$\begin{array}{cc} J_{f}^{S1}= & w_{L}P_{f}^{\mathrm{loss}}+w_{V}D_{V,f}+w_{\mathrm{viol}}C_{\mathrm{viol},f}+w_{G}G_{f}\\ \; & +w_{\mathrm{geo}}C_{\mathrm{geo},f}+C_{\mathrm{dup},f}-w_{\exp}E_{\exp,f}. \end{array}$$

This objective is a proposed study-specific scalar planning function. It combines feeder loss, voltage deviation, voltage-violation penalty, positive grid import, placement-suitability penalty, duplicate-allocation penalty, and bounded export reward. The loss term is accumulated over the selected months M and hours H using Equation (24):

(24) $$P_{f}^{\mathrm{loss}}=\sum_{m\in M}d_{m}\frac{24}{\left|H\right|}\sum_{h\in H}P_{f,m,h}^{\mathrm{loss}},$$

where $d_{m}$ is the daily weight associated with month *m*. Voltage quality is represented by Equation (25):

(25) $$D_{V,f}=\sum_{m\in M}d_{m}\frac{24}{\left|H\right|}\sum_{h\in H}\sum_{i\in B_{f}}{\left(|V_{i,m,h}|-1\right)}^{2}.$$

The voltage-violation penalty is defined in Equation (26):

(26) $$\begin{array}{cc} C_{\mathrm{viol},f}=\sum_{m,h,i}[ & \;\max{\left(V_{\min}-\left|V_{i,m,h}\right|,0\right)}^{2}\\ & \;+\max{\left(|V_{i,m,h}|-V_{\max},0\right)}^{2}]. \end{array}$$

The voltage limits are $V_{\min}=0.95\;p.u.$ and $V_{\max}=1.05\;p.u.$ The squared penalty increases the cost of severe voltage violations more strongly than small deviations. The corresponding code weights are $w_{L}=1$, $w_{V}=180$, $w_{\mathrm{viol}}=10^{6}$, $w_{G}=0.030$, and $w_{\mathrm{geo}}=45$. The intentionally dominant voltage penalty ensures that a low-loss but electrically infeasible solution is rejected.

Stage 2 minimizes the proposed cluster-level exchange objective in Equation (27):

(27) $$\begin{array}{cc} J^{S2}= & w_{G2}G_{\mathrm{rem}}+w_{\ell }L_{\mathrm{tie}}+w_{U}U-w_{R}R_{\mathrm{gain}}\\ \; & +w_{T}N_{\mathrm{tie}}+C_{\mathrm{conn}}-w_{E}E_{\mathrm{exch}}. \end{array}$$

Here, $G_{\mathrm{rem}}$ is residual import after exchange, $L_{\mathrm{tie}}=0.018E_{\mathrm{exch}}$ represents transfer loss, *U* is the unserved-dependency proxy, $R_{\mathrm{gain}}$ measures local supply improvement, $N_{\mathrm{tie}}$ is the number of closed ties, and $C_{\mathrm{conn}}$ penalizes disconnected exchange graphs. The weights are $w_{G2}=0.070$, $w_{\ell }=1.0$, $w_{U}=0.12$, $w_{R}=500$, and $w_{T}=25$; the adjustable exchange reward has default $w_{E}=0.030$. The value 0.018 represents a 1.8% planning-level tie-transfer loss allowance. This prevents Stage 2 from treating inter-feeder energy exchange as lossless. The objective does not include a full CAPEX/OPEX, battery-degradation, or net-present-value model. Its economic interpretation is therefore restricted to reduced grid dependence and reduced tie-transfer loss.

For each potential feeder pair (f,g), a normalized switch gene $x_{f,g}^{\mathrm{sw}}$ and capacity gene $x_{f,g}^{\mathrm{cap}}$ are decoded using Equations (28)–(30):

(28) $$\begin{array}{c} x_{f,g}^{\mathrm{sw}}>0.50\Rightarrow y_{f,g}=1, \end{array}$$

(29) $$\begin{array}{c} C_{f,g}=25+x_{f,g}^{\mathrm{cap}}C_{\max}, \end{array}$$

(30) $$\begin{array}{c} C_{\max}=\max\left(50,0.35P_{\mathrm{cluster}}^{\mathrm{peak}}\right). \end{array}$$

The threshold 0.50 is the normalized binary-decoding threshold for tie-switch closure. The offset of 25 kW prevents a selected tie from having negligible capacity. The value 50 kW is the minimum cluster-level exchange-capacity bound, while $0.35P_{\mathrm{cluster}}^{\mathrm{peak}}$ scales the maximum exchange capacity with the size of the feeder cluster. A disconnected exchange graph receives $C_{\mathrm{conn}}=5000$ to discourage infeasible transfer configurations.

### 2.7. GVFA Search, Constraint Handling, and Computational Procedure

Each GVFA goose corresponds to one candidate solution. The Stage 1 vector contains two variables for each resource: a location gene and a size gene. The Stage 2 vector contains switch and capacity genes for every possible feeder pair. Population members are initialized within [0,1], and the leader is the candidate with the lowest fitness. The GVFA is selected because the proposed planning problem is nonlinear, mixed discrete–continuous, nonconvex, and repeatedly constrained by BFS load-flow calculations. The location variables are discrete, the sizing variables are continuous, and the feasibility terms include clipping, voltage penalties, duplicate-bus correction, tie-switch decoding, and exchange-capacity constraints. These features make derivative-based optimization unsuitable, while a population-based derivative-free algorithm can search mixed decision spaces without requiring gradient information.

At iteration *t*, follower velocity is updated by inertia, attraction to the leader, alignment with the preceding goose, an asymmetric wing-offset term, and stochastic exploration, as expressed in Equation (31):

(31) $$\begin{array}{cc} v_{i}^{t+1}= & 0.55v_{i}^{t}+a_{t}\left(x_{L}^{t}-x_{i}^{t}\right)\\ \; & +b_{t}\left(x_{i-1}^{t}-x_{i}^{t}\right)+e_{t}w_{i}^{t}+g_{t}r_{i}^{t}. \end{array}$$

The inertia coefficient 0.55 preserves part of the previous velocity while limiting oscillation. The leader-attraction term guides candidates toward the current best solution, while the predecessor-following term represents the V-formation following mechanism. The wing-offset and random exploration terms preserve diversity.

The position update is given in Equation (32):

(32) $$x_{i}^{t+1}=\mathrm{clip}\left(x_{i}^{t}+v_{i}^{t+1},0,1\right).$$

The clipping operation ensures that all genes remain within the normalized decoding interval. With $\pi_{t}=t/\left(T_{\max}-1\right)$, the time-varying GVFA factors are defined in Equations (33)–(36):

(33) $$\begin{array}{cc} a_{t}=\alpha \left(1.15-0.35\pi_{t}\right),\; & \alpha =0.055, \end{array}$$

(34) $$\begin{array}{c} b_{t}=0.22\left(1-0.5\pi_{t}\right), \end{array}$$

(35) $$\begin{array}{c} e_{t}={\left(1-\pi_{t}\right)}^{1.35}, \end{array}$$

(36) $$\begin{array}{c} g_{t}=0.35\left(1-\pi_{t}\right)+0.05. \end{array}$$

The learning rate α=0.055 controls the strength of leader attraction and was selected to balance convergence speed against oscillatory movement. The term $1.15-0.35\pi_{t}$ gradually reduces leader attraction from early exploration to late exploitation. The coefficient 0.22 controls predecessor-following strength, while $1-0.5\pi_{t}$ reduces this influence as the search progresses. The exponent 1.35 in Equation (35) controls the nonlinear decay of the asymmetric wing-exploration term. The stochastic exploration schedule in Equation (36) decreases from early search to late search but retains a small value of 0.05 to avoid complete stagnation.

Every 25 iterations, the final goose is reinitialized to maintain diversity. Early stopping is activated after 18 stagnant iterations when the relative fitness improvement is below $10^{-5}$. These values are tuned algorithmic parameters. The reinitialization interval of 25 avoids excessive disruption of the population while reducing premature convergence. The patience value of 18 prevents termination due to short-term stochastic fluctuations, and the tolerance $10^{-5}$ stops the search only when improvement becomes negligible.

The GVFA parameters were selected through a two-level tuning strategy. First, a screening configuration was used to identify unstable or inefficient settings at moderate computational cost. Second, the final configuration was adopted for the reported simulations after verifying convergence stability, feasibility rate, final objective quality, and runtime. The final reported settings use 36 geese and 120 iterations in Stage 1 and 44 geese and 140 iterations in Stage 2. No theoretical superiority over PSO, GA, GWO, or other metaheuristics is claimed. Because the optimization problem is nonconvex and mixed discrete–continuous, global convergence cannot be guaranteed for GVFA or for the benchmark metaheuristics. The claim made in this study is empirical: under the same objective functions, search bounds, load-flow model, and comparable computational budgets, the proposed two-stage GVFA implementation provides stable planning solutions.

Constraint handling combines direct repair and penalty functions. All genes are clipped to [0,1] before decoding. Duplicate bus choices are repaired by assigning an unused high-suitability candidate where possible; residual duplicates are penalized. Electrical feasibility is not repaired heuristically because a local modification may affect the entire radial voltage profile. Instead, it is evaluated through BFS and strongly penalized through Equation (26). This design preserves the physical coupling among buses and avoids falsely treating voltage constraints as independent local restrictions.

**Figure 2:**
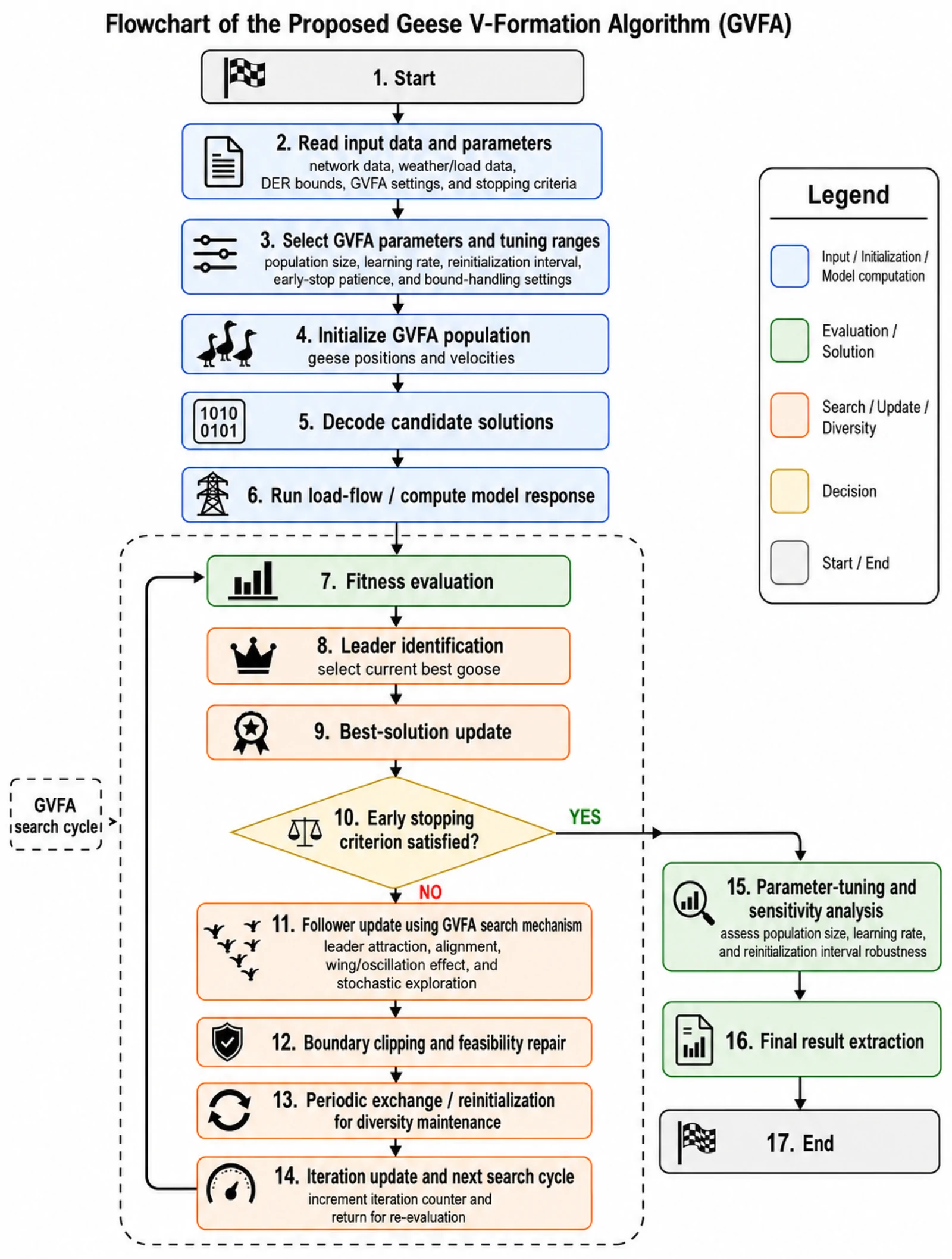
Flowchart of the proposed Geese V-Formation Algorithm (GVFA) used in the two-stage optimization framework.

**Figure 3:**
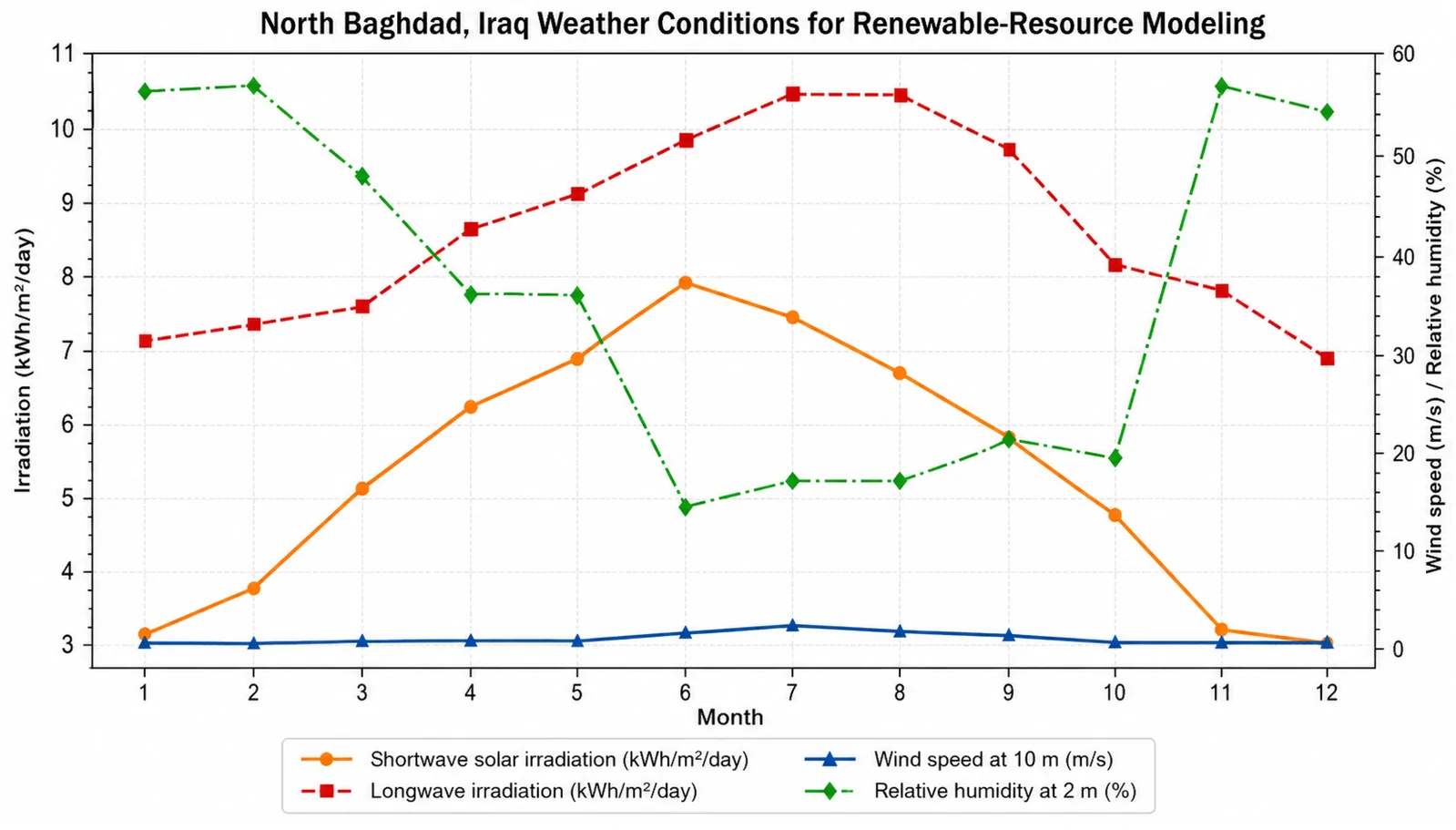
Monthly weather-driven PV, WT, and load factors used in the two-stage simulations.

The complete computational sequence follows the detailed workflow in Figure 1 and Flowchart of the proposed Geese V-Formation Algorithm (GVFA) used in the two-stage optimization framework in Figure 2: (i) read or construct feeder data; (ii) process NASA POWER weather records of Baghdad–Iraq, as shown in Figure 3; (iii) construct monthly PV, WT, diesel, and load multipliers; (iv) initialize and execute feeder-level GVFA in parallel; (v) create surplus/deficit profiles from the best Stage 1 portfolios; (vi) optimize ties and transfer capacities in Stage 2; (vii) conduct N-1, load-growth, measurement-input uncertainty, and GVFA parameter-sensitivity assessments; and (viii) write CSV, Excel, Word, and high-resolution graphical outputs. The two-stage structure is selected because the planning problem contains two coupled decision layers. Stage 1 determines local feeder-level DER and capacitor allocations under radial load-flow and voltage-security constraints. Stage 2 then uses the optimized feeder surplus–deficit profiles to select candidate tie-switch states and exchange capacities. This separation prevents the exchange layer from compensating for an electrically inadequate local resource portfolio and preserves the causal order between local adequacy and inter-feeder coordination. The complete computational implementation of this sequential structure is summarized in Algorithm 1, where Stage 1 first optimizes feeder-level DER and capacitor allocations, and Stage 2 subsequently uses the resulting surplus–deficit profiles to determine tie-switch states and exchange capacities.
**Algorithm 1** Two-stage weather-driven GVFA framework**Require:** Network data, weather data, DER bounds, GVFA parameters, M, H
**Ensure:** DER portfolios, tie-switch plan, losses, voltages, exchange, robustness results
  1:Build radial feeder models and weather multipliers  2:**for all** feeders *f* in parallel **do**  3:    Define PV×4, WT×1, BESS×4, CAP×2, and ${\mathrm{AUX}}_{f}$  4:    Initialize normalized Stage 1 GVFA population  5:    **while** termination criterion is not met **do**  6:        Decode locations and sizes for every goose  7:        Run BFS for all selected m∈M and h∈H  8:        Evaluate Equation (23); update leader and best solution  9:        Update followers using Equations (31)–(36); repair and clip genes10:        Reinitialize the final goose every 25 iterations11:    **end while**12:    Store the best Stage 1 portfolio and its net-grid profile13:**end for**14:Initialize Stage 2 GVFA for tie-switches and transfer capacities15:**while** termination criterion is not met **do**16:    Decode ties; simulate feeder exchange; evaluate Equation (27)17:    Update leader, followers, bounds, and periodic exchange state18:**end while**19:Run N-1 contingency, seasonal load-growth, measurement-input uncertainty, and GVFA parameter-sensitivity analyses20:Export numerical tables, figures, and final optimized solutions

### 2.8. GVFA Parameter-Sensitivity Analysis

To demonstrate the robustness of the GVFA implementation, a limited parameter-sensitivity analysis is included. This analysis does not attempt to prove theoretical convergence or universal superiority over other algorithms. Instead, it evaluates whether the final planning results are excessively dependent on a single parameter choice. The sensitivity analysis varies the most influential GVFA parameters while keeping the same feeder data, DER bounds, objective functions, BFS solver, voltage limits, and reporting metrics.

The sensitivity parameter set is defined in Equation (37):

(37) $$\Theta =\left\{N_{1},T_{1},N_{2},T_{2},\alpha ,T_{\mathrm{ex}},\varepsilon_{\mathrm{stop}},p,\eta_{\mathrm{tie}}\right\}.$$

For each sensitivity case *q*, the percentage deviation of a performance metric *Z* from the reference setting is calculated using Equation (38):

(38) $$\Delta Z_{q}=100\frac{Z_{q}-Z_{\mathrm{ref}}}{\left|Z_{\mathrm{ref}}\right|+\epsilon_{z}},$$

where $Z_{q}$ is the value obtained under sensitivity case *q*, $Z_{\mathrm{ref}}$ is the value obtained using the adopted reference setting, and $\epsilon_{z}$ is a small numerical safeguard. The reported metrics include final objective value, loss reduction, minimum voltage, voltage-violation probability, grid-import reduction, exchanged energy, convergence iteration, and runtime.

Because GVFA is stochastic, repeatability is evaluated using the coefficient of variation of the final best objective, as shown in Equation (39):

(39) $$CV_{J}=100\frac{\sigma \left(J_{\mathrm{best}}\right)}{\left|\mu \left(J_{\mathrm{best}}\right)\right|+\epsilon_{J}},$$

where $\mu (J_{\mathrm{best}})$ and $\sigma (J_{\mathrm{best}})$ are the mean and standard deviation of the final best objective over repeated runs. A lower $CV_{J}$ indicates more stable optimizer behavior.

To improve transparency in the GVFA implementation, Table 4 presents the algorithmic parameter-sensitivity protocol adopted for the proposed framework. The protocol identifies the principal parameters requiring sensitivity assessment, including Stage-1 and Stage-2 population sizes, iteration limits, learning rate, reinitialization interval, early-stopping settings, tie-loss factor, and random-seed repeatability. It also defines the corresponding performance indicators, including final objective value, loss reduction, minimum voltage, runtime, exchanged energy, residual grid import, tie-loss term, feasibility rate, stagnation behavior, and coefficient of variation. However, Table 4 should be interpreted as a protocol-level transparency addition rather than a completed numerical sensitivity analysis. Therefore, no formal parameter-robustness claim is made unless the corresponding numerical sensitivity results are generated and reported.

### 2.9. Simulation Settings, Complexity, and Implementation Scope

Table 5 summarizes the principal optimization settings used in the final simulations. The reported results were obtained using the complete weather-driven configuration, including all 24 hourly operating points, 36 geese and 120 iterations in Stage 1, and 44 geese and 140 iterations in Stage 2. A fixed random seed of 42 was adopted to ensure reproducibility. These values provide a practical balance between search diversity and the computational burden associated with repeated radial load-flow evaluations.

Let *F* be the number of feeders, $N_{1}$ the Stage 1 population, $T_{1}$ the Stage 1 iterations, *M* the evaluated months, *H* the evaluated hours, and $C_{\mathrm{BFS}}$ the cost of one load-flow evaluation. The leading Stage 1 complexity is defined in Equation (40):

(40) $$O\left(FN_{1}T_{1}MHC_{\mathrm{BFS}}\right).$$

With *W* parallel workers, the practical workload is approximated by Equation (41):

(41) $$O\left(\left\lceil \frac{F}{W}\right\rceil N_{1}T_{1}MHC_{\mathrm{BFS}}\right).$$

For Stage 2, $E_{p}=F\left(F-1\right)/2$ possible feeder pairs yield Equation (42):

(42) $$O\left(N_{2}T_{2}MHE_{p}\right),$$

which is typically lower because Stage 2 uses feeder-level import/export matrices rather than repeated bus-level BFS calculations. The primary memory requirement is $O(N_{1}D_{1}+N_{2}D_{2}+F|B|H)$, where $D_{1}$ and $D_{2}$ are the Stage 1 and Stage 2 decision dimensions.

The framework is implemented in Python 3.13 using NumPy, Pandas, Matplotlib, argparse, dataclasses, pathlib, itertools, random, warnings, and concurrent.futures. Parallel feeder optimization uses ProcessPoolExecutor; a worker value of zero selects the available CPU count, while a positive value fixes the number of worker processes. The BFS solver and connectivity check are implemented directly, the latter through depth-first search.

### 2.10. Implementation Scope and Limitations

The proposed framework is intended for planning-stage DER allocation and feeder-coordination assessment. It does not claim direct real-time operational control or field-ready DERMS deployment. The BESS model uses a prescribed normalized flexibility profile and does not include chronological SOC evolution, energy-capacity optimization, charging/discharging efficiency, degradation, cycle ageing, converter dynamics, replacement cost, or market-based dispatch. Therefore, BESS-related results should be interpreted as planning-level network-support effects.

The BFS load-flow model assumes balanced steady-state radial operation. Consequently, unbalanced three-phase operation, harmonics, transient stability, detailed protection coordination, communication latency, and real-time control constraints are outside the present scope. As clarified in Table 2, practical implementation would require synchronized measurements from AMI, SCADA/DMS, DER inverter telemetry, BMS, weather sensors, tie-switch status devices, and tie-line meters. Future work should extend the framework to unbalanced three-phase feeder models, time-coupled SOC-constrained BESS optimization, charging/discharging efficiency modeling, degradation-aware storage planning, detailed thermal and protection constraints, real-time DERMS integration, and validation using synchronized field measurements.

## 3. Results and Discussion

**Figure 4:**
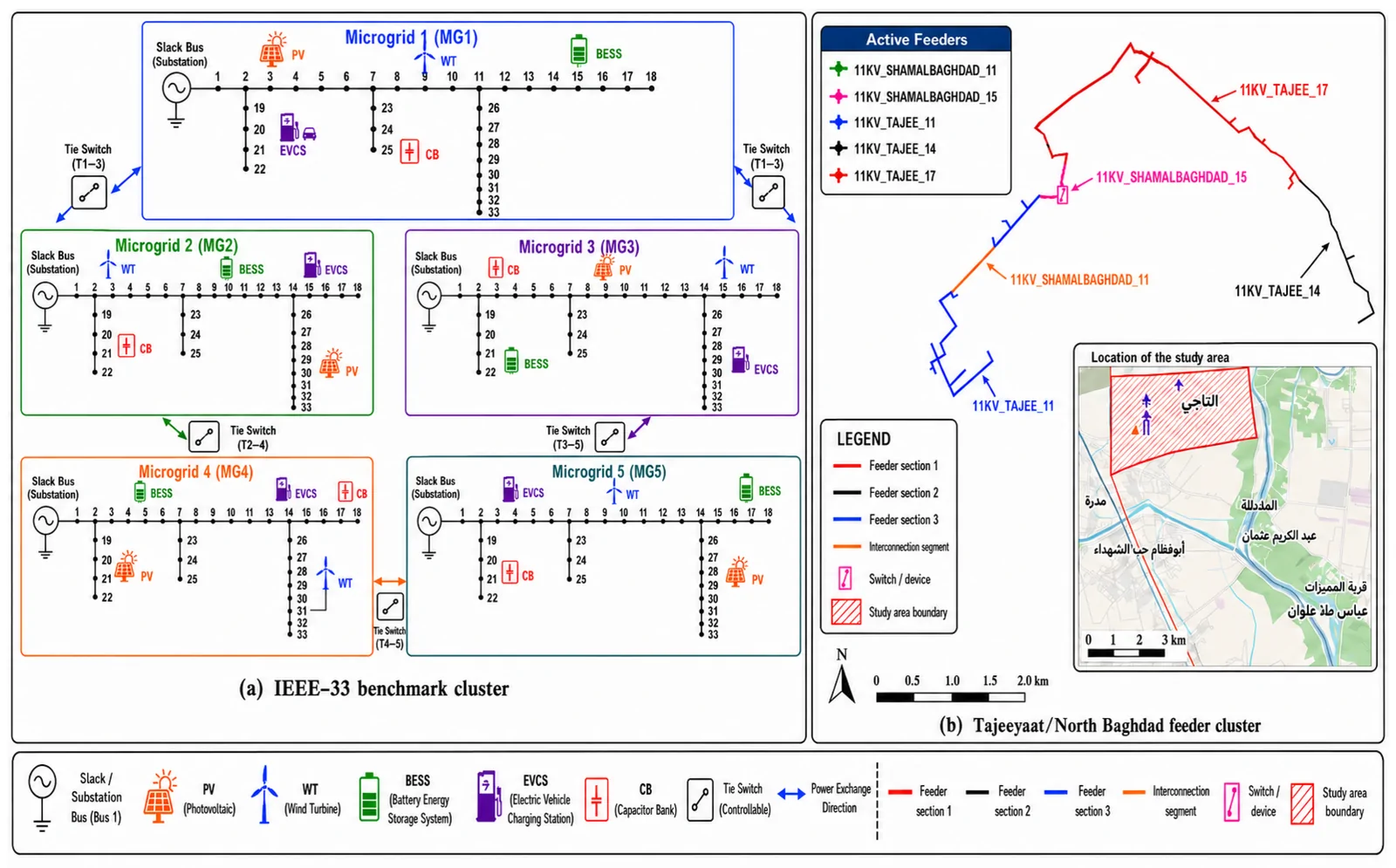
IEEE-33 benchmark and a practical five-feeder 11 kV Tajeeyaat/North Baghdad distribution clusters. *Note:* Numbers in panel (**a**) denote bus indices; the Arabic term (non-English) in the inset map denotes Al-Taji, the study-area location.

This section evaluates the proposed weather-driven two-stage Geese V-Formation Algorithm (GVFA) using a five-instance IEEE-33 benchmark cluster and a practical five-feeder 11 kV Tajeeyaat/North Baghdad distribution cluster, as shown in Figure 4. The discussion integrates feeder-level DER placement results, active-loss reduction, voltage regulation, Stage-2 energy exchange, weather dependence, contingency response, seasonal demand growth, and normalized convergence behavior. The interpretation is deliberately based on radial-network physics: reduction in upstream active and reactive current lowers I^2^R losses; local photovoltaic (PV), wind-turbine (WT), battery energy storage system (BESS), and capacitor-banks injections improve voltage magnitude by reducing the effective power-transfer distance; and Stage-2 exchange redistributes local surplus subject to tie capacity and network losses. The results should be interpreted as balanced steady-state planning results. Table 6 establishes two distinct operating contexts. The IEEE-33 cluster begins from a severe low-voltage condition, with feeder minimum voltages between 0.8268 and 0.8632 p.u., whereas the practical cluster starts from a comparatively higher range of 0.9497–0.9898 p.u. After Stage-1 optimization, the aggregate loss reduction is 79.90% for the benchmark and 70.09% for the practical cluster. The different post-optimization voltage margins are important for interpreting the robustness tests: the practical system retains a wider voltage-security margin, while the stressed benchmark exposes its hosting-capacity boundary under seasonal demand growth. Table 7 summarizes the optimal locations and installed capacities of PV, WT, BESS, and auxiliary resources for the IEEE-33 benchmark cluster and the Tajeeyaat/North Baghdad 11-kV feeders. The results confirm that the GVFA allocates diversified DER portfolios at electrically suitable buses to strengthen local supply, support voltage regulation, and create feasible surplus capacity for inter-feeder energy exchange.

Table 7 reports the optimized Stage-1 DER portfolio for each feeder. Each cell first gives the total installed capacity of the corresponding technology within that feeder, followed by the selected bus or node locations and their individual power ratings. For example, an entry such as “Total = 4155.6; B02: 1855.7; B27: 960.3” means that the total PV capacity installed in the feeder is 4155.6 kW, distributed across Bus 2 and Bus 27 with the listed capacities. For the IEEE-33 benchmark, labels of the form Bxx denote Bus xx. For the practical Tajeeyaat/North Baghdad system, the original utility node identifiers are retained to preserve traceability to the utility dataset. The BESS values in this table represent optimized power ratings in kW; they should not be interpreted as energy capacity or chronological battery-dispatch results.

### 3.1. Feeder-Level Loss Reduction and Voltage Regulation

**Figure 5:**
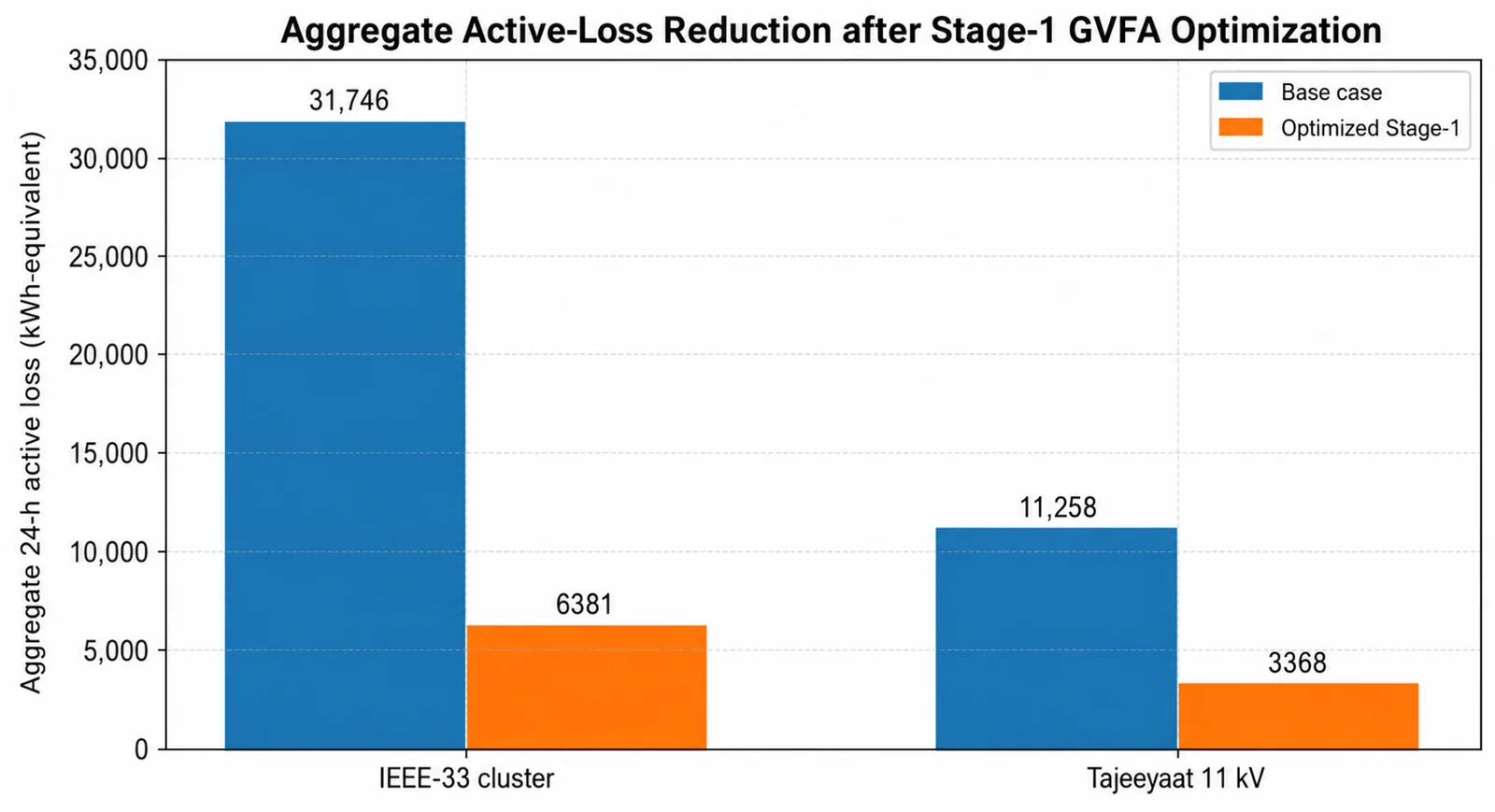
Aggregate 24 h active-loss comparison before and after Stage-1 GVFA optimization for the IEEE-33 benchmark and the Tajeeyaat/North Baghdad feeder cluster.

As illustrated in Figure 5, the coordinated placement of PV, WT, BESS, capacitor banks, and auxiliary resources substantially reduces the aggregate active-loss requirement in both cases. This behavior follows the quadratic current-loss mechanism of radial feeders. Local active-power injection reduces upstream current, whereas capacitor-bank and inverter/BESS reactive support reduce the reactive component of branch current. The simultaneous reduction in both current components explains why the optimization produces markedly larger loss improvements than a placement strategy based on active power alone. The result is physically credible because the algorithm concentrates flexible resources near electrically influential buses, thereby reducing the downstream current that would otherwise traverse high-impedance paths.

**Figure 6:**
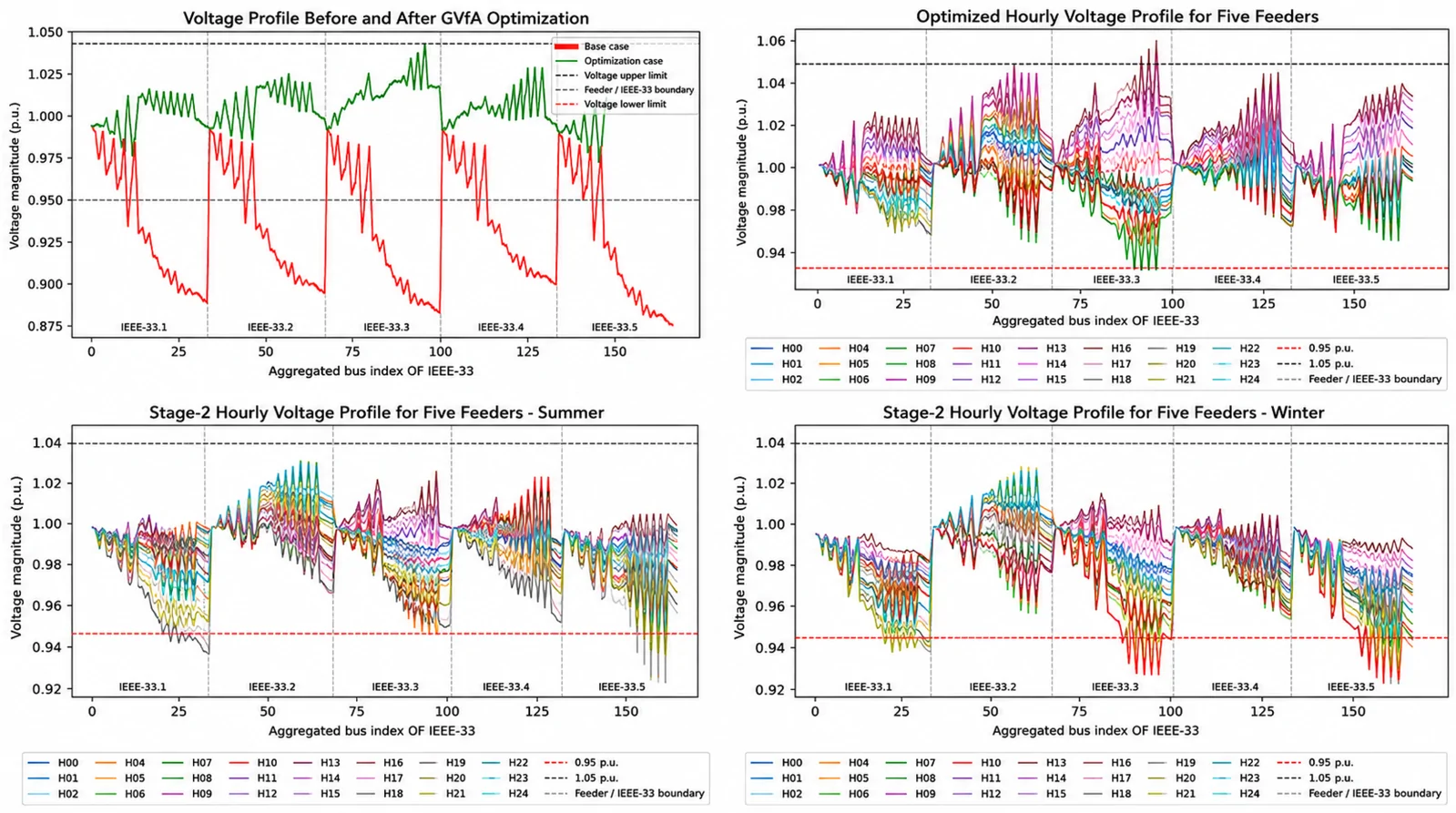
Feeder-level voltage profile improvement for IEEE-33 system.

As illustrated in Figure 6 and quantified in Table 8, the GVFA-based Stage-1 optimization substantially improves the electrical performance of all five interconnected IEEE-33 feeders by simultaneously reducing feeder losses and recovering the voltage profile from critically undervoltage base-case conditions. The base-case minimum voltages range from 0.8268 p.u. in IEEE33_FEEDER_5 to 0.8632 p.u. in IEEE33_FEEDER_4, confirming severe voltage weakness before coordinated DER allocation; after optimization, the minimum voltages increase to 0.9642, 0.9606, 0.9465, 0.9683, and 0.9612 p.u. for Feeders 1–5, respectively, where the optimized voltage trajectories are shifted upward toward the nominal range and remain predominantly bounded by the prescribed 0.95–1.05 p.u. operating limits across the aggregated feeder system. In parallel, daily loss reductions range from 71.56% for IEEE33_FEEDER_2 to 85.03% for IEEE33_FEEDER_4, reducing the highest base loss of 7818.89 kWh-eq/24 h in IEEE33_FEEDER_5 to 1455.50 kWh-eq/24 h. The corresponding grid-import reductions of 64.95–95.40% demonstrate that the optimized PV, WT, BESS, EVCS, and capacitor-bank deployment materially increases local energy utilization and reduces upstream dependency. The hourly summer and winter voltage profiles in Figure 6 further indicate that the coordinated operating strategy preserves acceptable voltage regulation under time-varying loading and renewable-generation conditions; however, the Stage-1 minimum voltage of IEEE33_FEEDER_3 remains marginally below 0.95 p.u., identifying this feeder as the principal candidate for additional Stage-2 inter-feeder support, reactive-power reinforcement, or refined DER/BESS dispatch.

**Figure 7:**
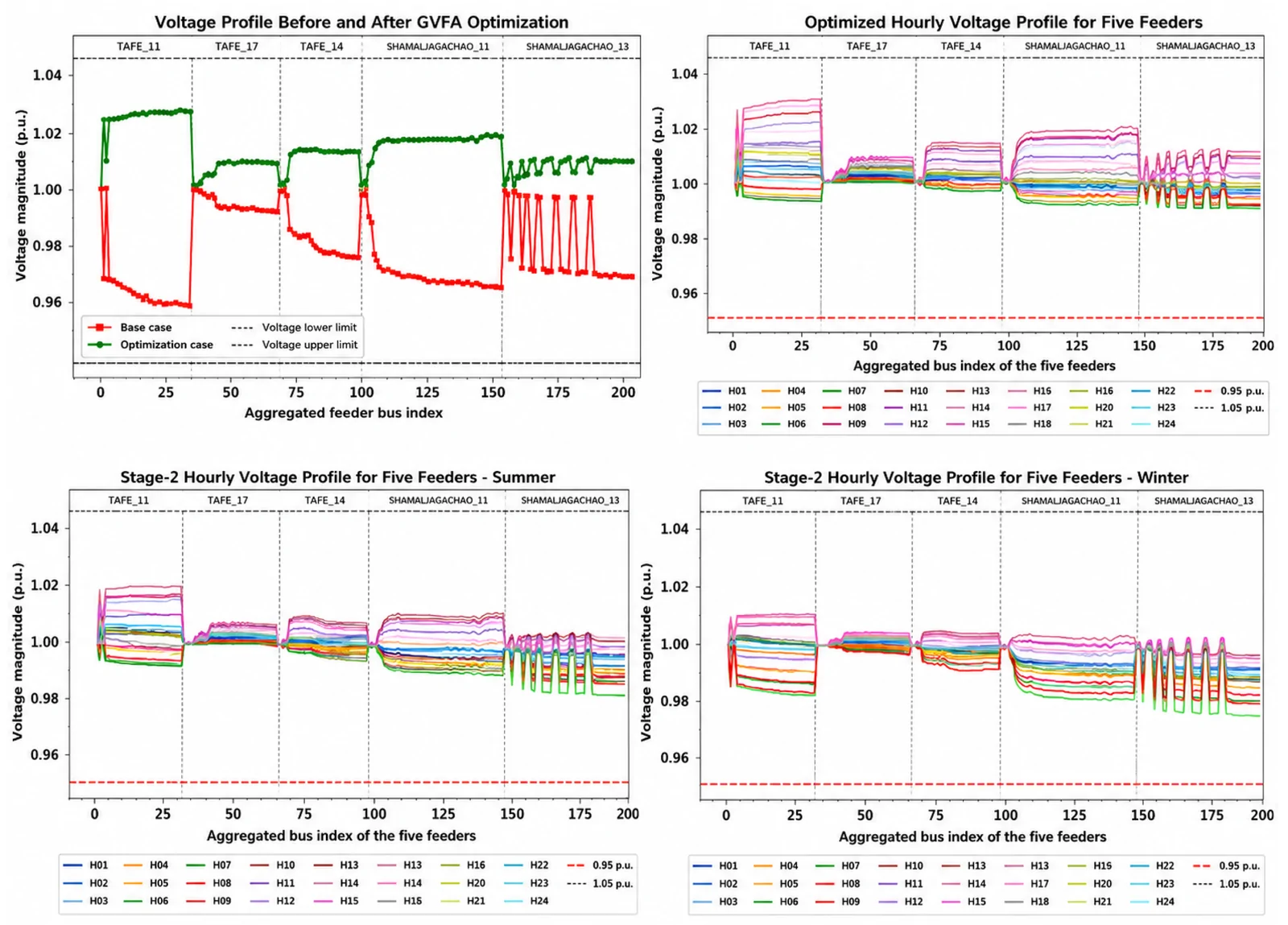
Feeder-level voltage profile improvement for Tajeeyaat/North Baghdad cluster.

As demonstrated in Figure 7 and quantified in Table 9, the proposed GVFA-based Stage-1 scheduling markedly strengthens voltage regulation and reduces feeder losses across the Tajeeyaat/North Baghdad cluster. The optimized profiles shift all feeders away from the 0.95 p.u. lower operating limit, increasing the minimum voltage from 0.9497–0.9898 p.u. in the base condition to 0.9897–0.9997 p.u. after optimization; this improvement remains robust across the hourly, summer, and winter voltage trajectories shown in Figure 7. The largest loss reductions are obtained for 11KV_SHAMALBAGHDAD_11 (83.16%), 11KV_SHAMALBAGHDAD_15 (79.38%), and 11KV_TAJEE_14 (68.43%), while 11KV_TAJEE_11 achieves a 62.51% reduction. These improvements are accompanied by substantial grid-import reductions of 87.79–97.37% for four feeders, confirming more effective local utilization of distributed resources and reduced dependence on upstream supply. Although 11KV_TAJEE_17 exhibits a limited 7.63% increase in feeder loss, its minimum voltage improves from 0.9898 to 0.9997 p.u., and its grid import is reduced by 99.69%; therefore, this result should be interpreted as a controlled operational trade-off associated with voltage support and local energy balancing rather than an overall degradation in feeder performance. Overall, the combined numerical and graphical results confirm that the proposed coordination strategy improves voltage security while achieving significant loss and grid-import reductions under temporally varying operating conditions.

### 3.2. Stage-2 Inter-Feeder Exchange and Bidirectional Power-Flow Implications

The Stage 2 results demonstrate the main function of the proposed hierarchical framework: after each feeder is locally optimized in Stage 1, the remaining surplus and deficit profiles are used to coordinate energy exchange at the feeder-cluster level. This transforms the feeder set from electrically isolated optimized units into a cooperative local energy-sharing cluster. Positive net exchange represents feeder export, whereas negative net exchange denotes imported support. Therefore, the reported exchange values quantify the residual energy surplus or deficit after accounting for local PV and WT generation, BESS flexibility, capacitor-bank support, auxiliary resources, and feeder demand.

The engineering significance of Stage 2 is that it does not assume energy sharing as an automatic benefit. Instead, it identifies which feeders can provide surplus energy, which feeders remain dependent on external support, and which tie corridors become technically important for local balancing. This provides a practical planning advantage over isolated feeder-level DER allocation because local DER portfolios may reduce losses and improve voltages within individual feeders while still leaving unbalanced surplus and deficit conditions across the feeder group. By using the optimized feeder boundary profiles, Stage 2 provides a structured mechanism for selecting tie-switch states and exchange capacities that can reduce residual grid dependence and improve local utilization of available DER energy.

**Figure 8:**
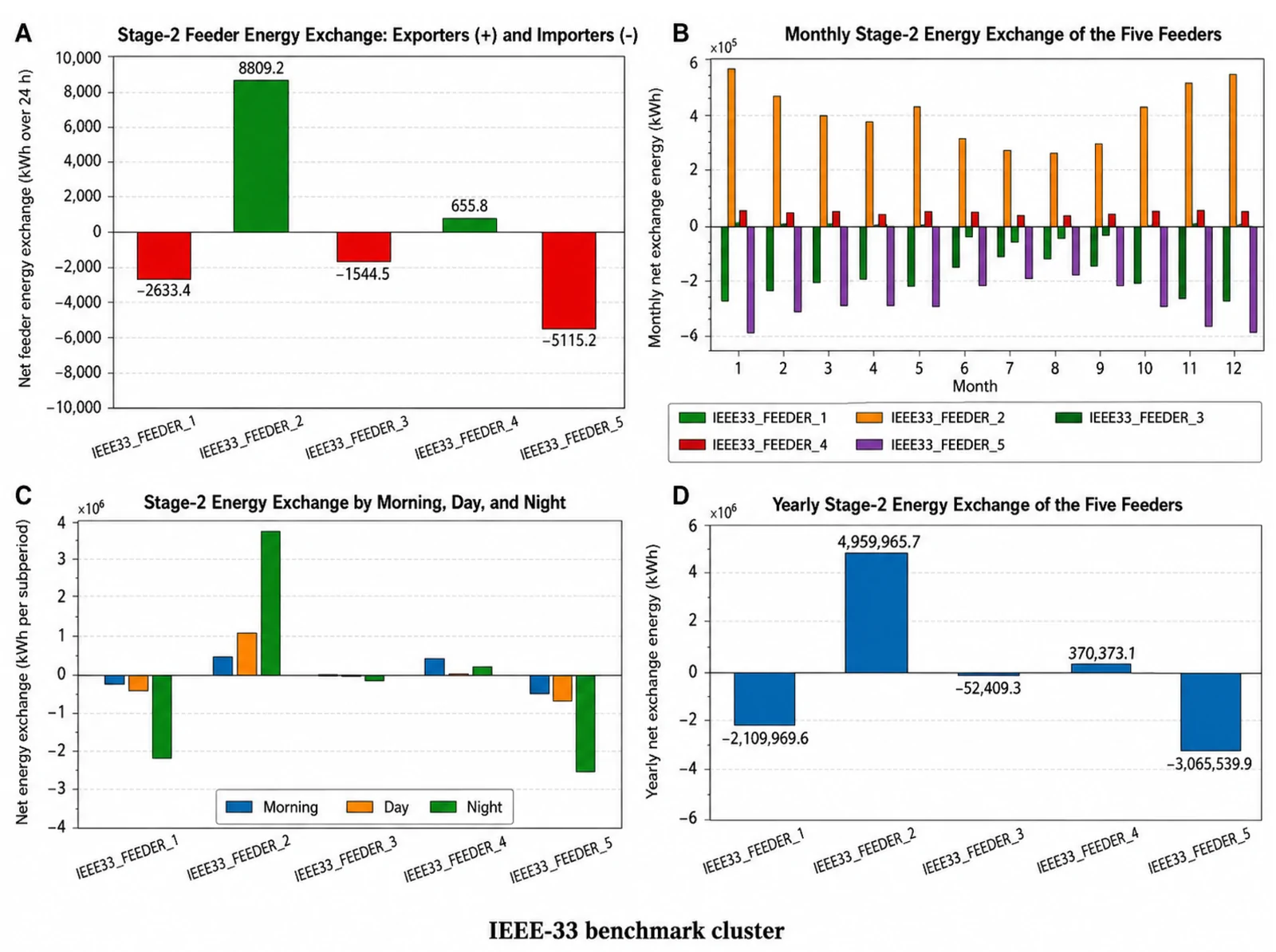
Stage-2 energy exchange among the five IEEE-33 benchmark feeder instances.

For the IEEE-33 benchmark cluster, Table 10 and Table 11, together with Figure 8, show a clear exporter–importer structure over the evaluated 24 h period. IEEE33_FEEDER_2 is the dominant exporter, supplying 8809.2 kWh, while IEEE33_FEEDER_4 exports 655.8 kWh. Conversely, IEEE33_FEEDER_1, IEEE33_FEEDER_3, and IEEE33_FEEDER_5 import 2633.4 kWh, 1544.5 kWh, and 5115.2 kWh, respectively. Total exports reach 9465.0 kWh, compared with 9293.1 kWh of total imports. The residual difference of 171.9 kWh, equivalent to approximately 1.82% of gross exported energy, is consistent with the planning-level exchange-loss allowance and boundary-energy accounting. This close balance supports the physical coherence of the Stage 2 coordination results.

The distribution of exchange is not uniform. IEEE33_FEEDER_2 contributes approximately 93.1% of the total exported energy, while IEEE33_FEEDER_5 accounts for approximately 55.0% of total imports. This concentration indicates that the transfer corridors associated with these feeders are the most important interfaces in the IEEE-33 cluster. From an engineering perspective, such corridors should be prioritized for tie-switch controllability, current-loading verification, voltage-limit checking, and reverse-power protection assessment. The result also shows that energy balancing alone is insufficient; a feasible exchange plan must remain compatible with AC power-flow behavior, branch-capacity limits, voltage constraints, and protection requirements.

The monthly and yearly results confirm that the IEEE-33 exchange structure is not limited to a single operating day. IEEE33_FEEDER_2 remains the major annual exporter, with 4.960 GWh, followed by IEEE33_FEEDER_4 with 0.370 GWh. IEEE33_FEEDER_5 is the largest annual importer at 3.070 GWh, followed by IEEE33_FEEDER_1 at 2.110 GWh. IEEE33_FEEDER_3 remains nearly energy-neutral, with a relatively small annual import of 0.052 GWh. These findings indicate that Stage 2 can identify persistent feeder roles across time, which is useful for planning normally open tie locations, feeder reinforcement priorities, and monitoring requirements.

The time-of-day exchange results provide additional insight. The largest transfers occur during the night period, particularly between the dominant exporting and importing feeders. This pattern indicates that Stage 2 coordination is not governed only by daytime photovoltaic output. Instead, it is influenced by load diversity, wind availability, BESS flexibility, residual demand, and the timing of local surplus or deficit. Consequently, a fixed exchange assumption would be inadequate for practical operation. Although the present study is a planning-level analysis, future implementation would require time-resolved tie-switch supervision and feeder-monitoring data to ensure that instantaneous flows remain within voltage, current, and protection limits.

**Figure 9:**
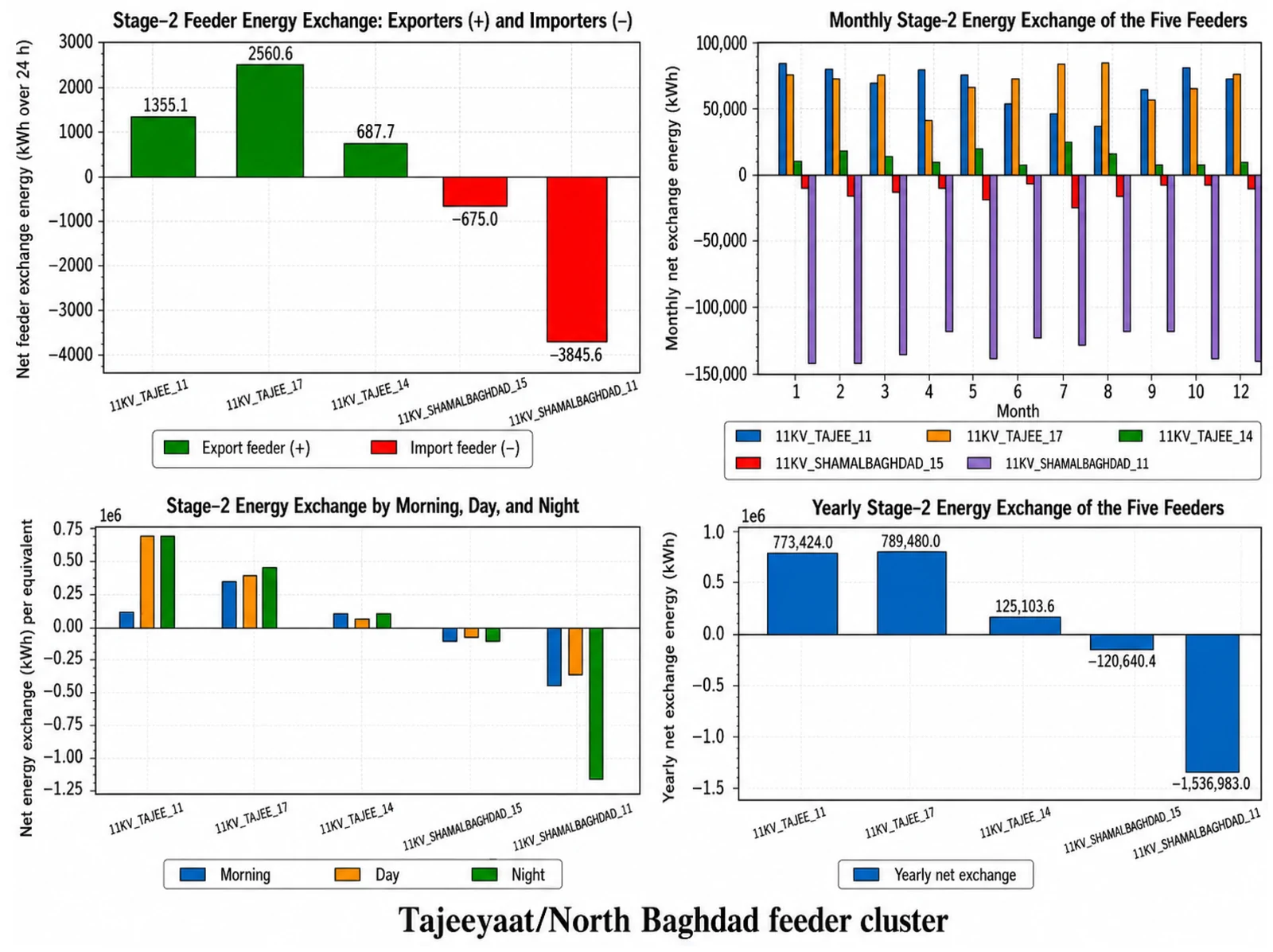
Stage-2 energy exchange among the five feeders in each cluster of Tajeeyaat.

A similar but geographically realistic exchange pattern is observed for the Tajeeyaat/North Baghdad feeder cluster. As shown in Table 10 and Table 11, together with Figure 9, 11KV_TAJEE_11, 11KV_TAJEE_17, and 11KV_TAJEE_14 act as net exporters over the evaluated 24 h period, exporting 1355.1 kWh, 2560.6 kWh, and 687.7 kWh, respectively. In contrast, 11KV_SHAMALBAGHDAD_15 and 11KV_SHAMALBAGHDAD_11 act as importing feeders, with imports of 675.0 kWh and 3845.6 kWh. Total exports are 4603.4 kWh, while total imports are 4520.6 kWh, leaving a residual of 82.8 kWh, or approximately 1.80% of gross exports. This result is consistent with the planning-level tie-loss representation and confirms that the exchange formulation preserves a coherent energy balance in the practical feeder cluster.

The real-network results identify a strong dependence on the Tajee feeders, particularly 11KV_TAJEE_17, which provides approximately 55.6% of total daily export. Meanwhile, 11KV_SHAMALBAGHDAD_11 absorbs approximately 85.1% of total imported energy, identifying it as the principal deficit feeder. This relationship remains evident at the annual scale: 11KV_TAJEE_11, 11KV_TAJEE_17, and 11KV_TAJEE_14 export 0.773 GWh, 0.789 GWh, and 0.125 GWh, respectively, whereas 11KV_SHAMALBAGHDAD_15 and 11KV_SHAMALBAGHDAD_11 import 0.121 GWh and 1.537 GWh, respectively. These results have direct planning implications. The Tajee feeders represent potential local support sources, whereas 11KV_SHAMALBAGHDAD_11 should be considered a priority feeder for voltage monitoring, capacity reinforcement, local DER expansion, or controlled tie support.

The selected tie-switch configurations in Table 10 show that the proposed Stage 2 method identifies practical exchange corridors rather than relying on a fully meshed or unrestricted exchange assumption. In the IEEE-33 benchmark cluster, the largest selected tie capacity is between IEEE33_FEEDER_4 and IEEE33_FEEDER_5, while strong corridors also connect IEEE33_FEEDER_1–IEEE33_FEEDER_2 and IEEE33_FEEDER_2–IEEE33_FEEDER_5. In the Tajeeyaat/North Baghdad case, the strongest selected corridors connect 11KV_TAJEE_14 with 11KV_SHAMALBAGHDAD_15 and 11KV_TAJEE_17 with 11KV_SHAMALBAGHDAD_11. These selected ties identify where controllable switching, protection review, tie-line metering, and thermal-capacity verification would be most important in a practical implementation.

The monthly and time-segmented profiles also show that feeder roles are broadly stable, but exchange magnitudes vary according to seasonal demand and renewable availability. Therefore, annual or monthly net exports should not be interpreted as fixed instantaneous power-flow directions. A feeder that is a net annual exporter may temporarily import power during peak-demand or low-generation intervals. The inter-feeder interfaces must therefore permit bidirectional power transfer, with the instantaneous flow direction determined by time-resolved demand, renewable output, BESS flexibility, feeder loading, voltage limits, and the active switching configuration.

These results highlight three key engineering findings. First, Stage 2 converts independently optimized feeders into a coordinated local energy-sharing cluster, thereby improving the utilization of feeder-level DER surplus. Second, the exchange-balance residuals of approximately 1.82% for IEEE-33 and 1.80% for Tajeeyaat/North Baghdad are consistent with the planning-level tie-loss representation, supporting the physical consistency of the exchange calculation. Third, the dominant exporter–importer pairs identify practical priority corridors for monitoring, protection coordination, and reinforcement planning. Accordingly, the proposed Stage 2 formulation provides not only a numerical reduction in residual grid dependence but also a planning map of where feeder-to-feeder exchange infrastructure would be most valuable.

Nevertheless, the Stage 2 results should be interpreted as planning-level evidence, not as a complete real-time operating strategy. Practical deployment would require synchronized feeder measurements, tie-switch status monitoring, bidirectional metering, thermal ratings, voltage-security verification, and protection-coordination studies. Within this planning scope, however, the results demonstrate that the proposed Stage 2 layer can identify technically meaningful exchange paths, quantify feeder surplus and deficit roles, and support more informed decisions regarding inter-feeder coordination in active distribution networks.

### 3.3. N-1 Contingency Performance and Resilience Interpretation

**Figure 10:**
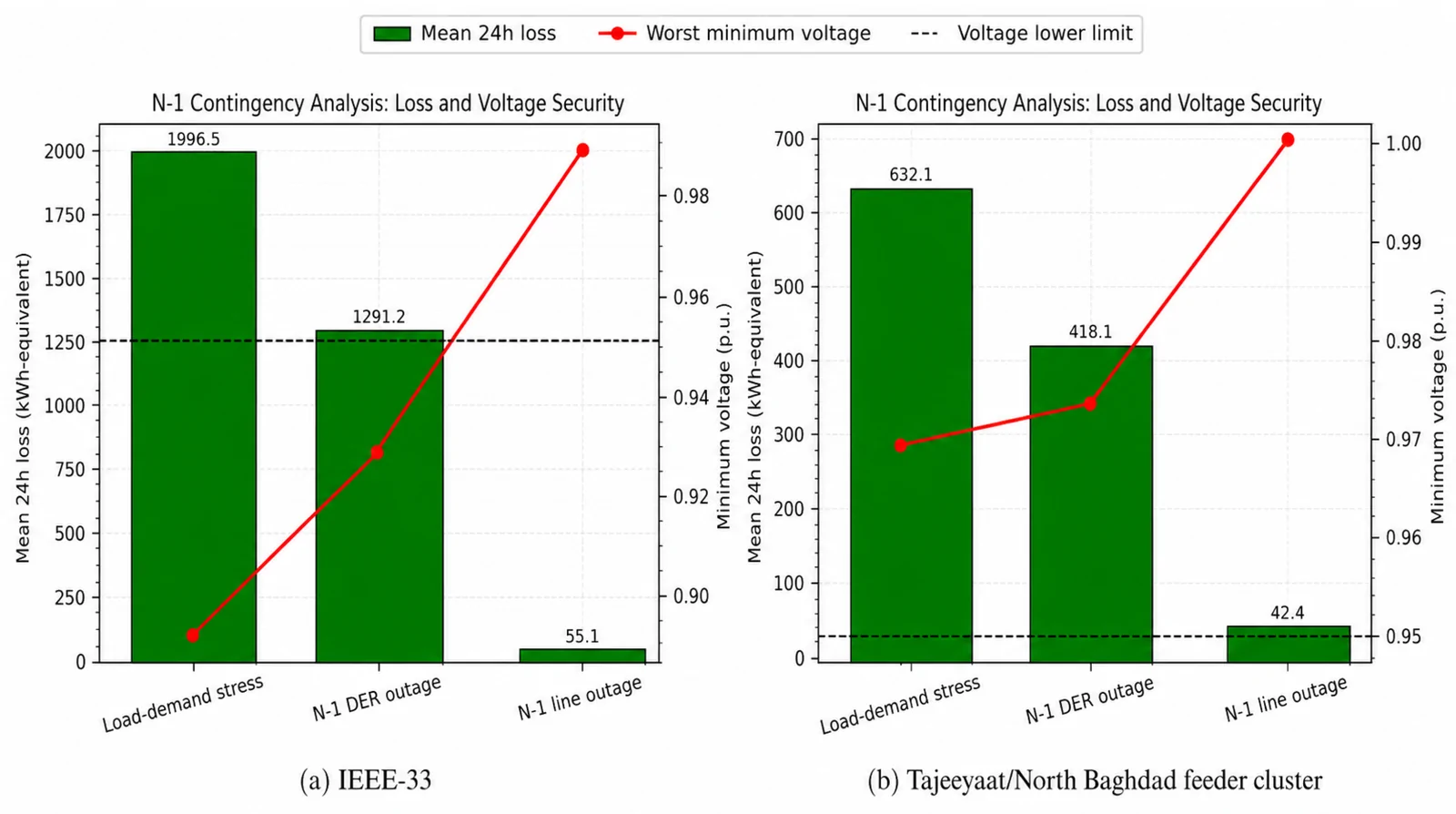
Aggregate N-1 contingency comparison: mean 24 h loss and worst minimum voltage by event class.

Figure 10 and Table 12 distinguish between two different aspects of contingency performance: voltage security of the energized network and continuity of supply to all loads. This distinction is essential for interpreting the line-outage results. In the N-1 line-outage cases, removing a branch may electrically isolate downstream buses from the slack bus. The loads connected to these isolated buses are counted as energy not served (ENS). However, because these buses are no longer energized, they are not included in the voltage-limit assessment performed by the BFS load-flow solution. Therefore, the reported minimum voltage and the “Any voltage violation” indicator refer only to buses that remain energized and connected to the slack bus after the contingency.

This explains the apparently contradictory IEEE-33 line N-1 result in Table 12. The worst energized-bus minimum voltage remains 0.9893 p.u., and therefore no voltage violation is reported for the supplied portion of the network. At the same time, the maximum ENS reaches 90,558 kWh, equivalent to approximately 90.56 MWh, because a downstream section is disconnected and its load is not supplied. Hence, the case should not be interpreted as fully resilient; rather, it indicates that the energized portion remains voltage-secure while the feeder experiences a significant service-continuity problem.

The same interpretation applies to the Tajeeyaat/North Baghdad line-outage case, where the energized portion remains above the voltage limit but a higher maximum ENS is observed due to disconnected load. Conversely, DER-outage and demand-stress scenarios do not disconnect buses; therefore, their ENS is zero, and their severity is reflected mainly through voltage degradation and loss increase. In the IEEE-33 cluster, DER outages and demand-stress scenarios cause lower-voltage violations, with the aggregate worst minimum voltage falling below the 0.95 p.u. threshold. This demonstrates that normal-operation improvement alone does not establish all-season N-1 adequacy. Conversely, the Tajeeyaat/North Baghdad cluster remains above the voltage limit in all tested contingency classes, reflecting the stronger base voltage margin and the spatial diversification of PV, WT, BESS, and capacitor placements.

The low losses reported for line-outage cases should not be interpreted as improved efficiency. Line isolation can reduce calculated feeder losses because disconnected downstream loads no longer draw current from the supplied network. Therefore, reduced losses under a line outage may indicate unsupplied demand rather than improved operational performance. The correct resilience interpretation requires voltage security, power losses, and ENS to be considered jointly. A contingency with no voltage-limit violation but high ENS should be classified as voltage-secure for the energized portion of the network but supply-continuity critical for the disconnected load section.

### 3.4. Seasonal Load-Growth Sensitivity and Hosting-Capacity Boundary

**Figure 11:**
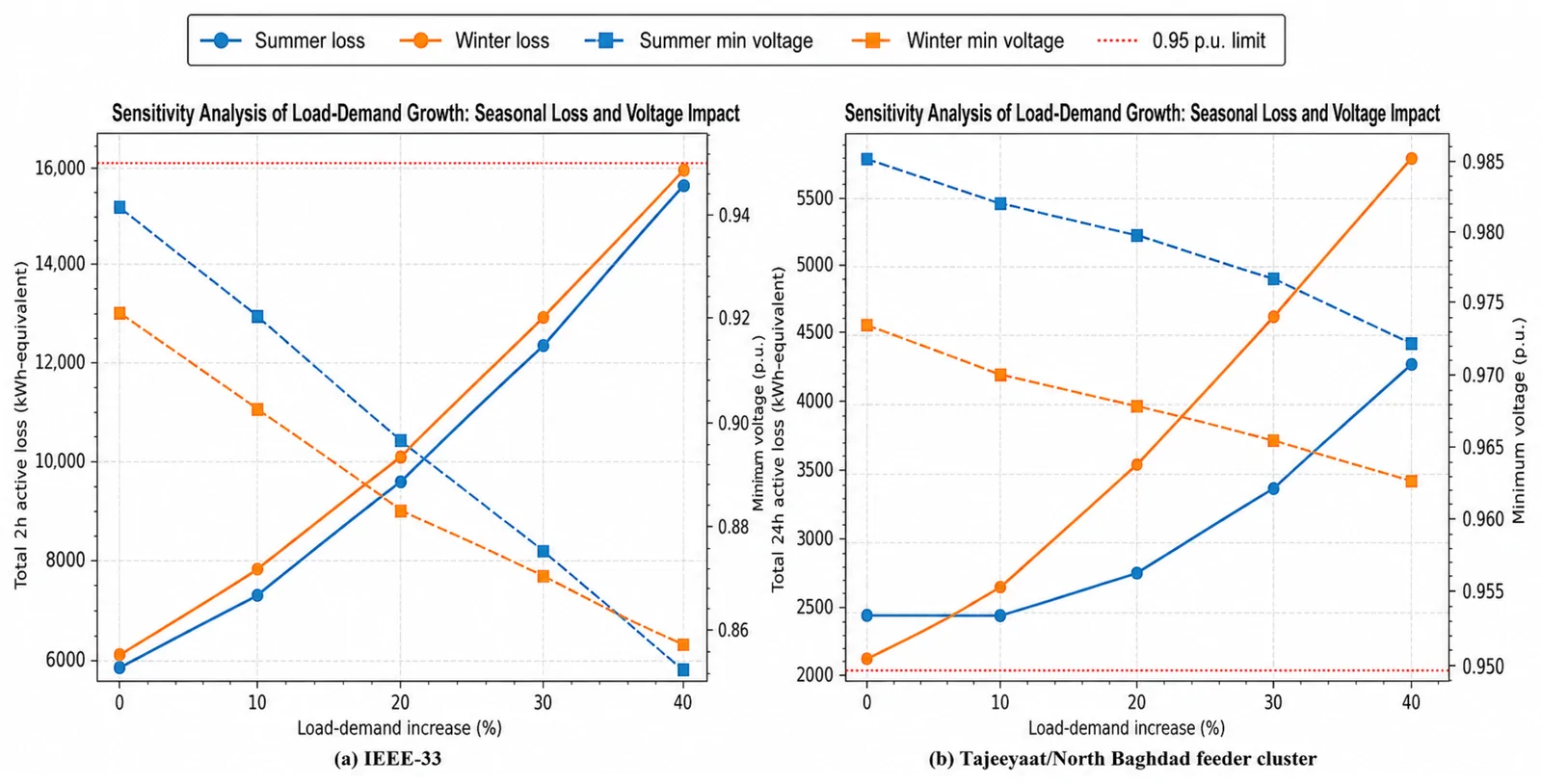
Seasonal load-growth sensitivity: loss growth and minimum-voltage response for both case studies.

The seasonal load-growth sensitivity results, as reported in Table 13 and illustrated in Figure 11, show clearly different robustness levels for the benchmark and practical networks. In the IEEE-33 cluster, increasing demand from 0% to 40% raises total 24 h losses from 5967.11 to 15,860.67 kWh-equivalent in summer and from 6185.57 to 16,128.57 kWh-equivalent in winter. Simultaneously, the minimum voltage drops from 0.9251 to 0.8539 p.u. in summer and from 0.9294 to 0.8657 p.u. in winter, remaining below the 0.95 p.u. threshold for all growth levels. Conversely, the Tajeeyaat/North Baghdad feeders maintain voltage compliance under the same stress, although losses and grid imports increase monotonically. At 40% growth, the practical system keeps minimum voltages of 0.9698 p.u. in summer and 0.9625 p.u. in winter. The figure confirms these trends, highlighting the practical feeder cluster’s stronger voltage-security margin and its superior hosting capability under seasonal demand escalation compared with the benchmark case.

### 3.5. Convergence Characteristics and Computational Interpretation

The optimizer comparison was conducted under identical problem settings for all methods, including the same feeder cases, decision-variable encoding, population size, iteration limit, random seed values, DER constraints, voltage limits, weather inputs, and Stage 1 objective-function formulation. GVFA, PSO, GA, and random search were independently implemented in Python and evaluated through the same objective-function routine. Therefore, all candidate solutions were assessed using the same technical indices, voltage penalties, placement constraints, and weather-dependent operating conditions.

**Figure 12:**
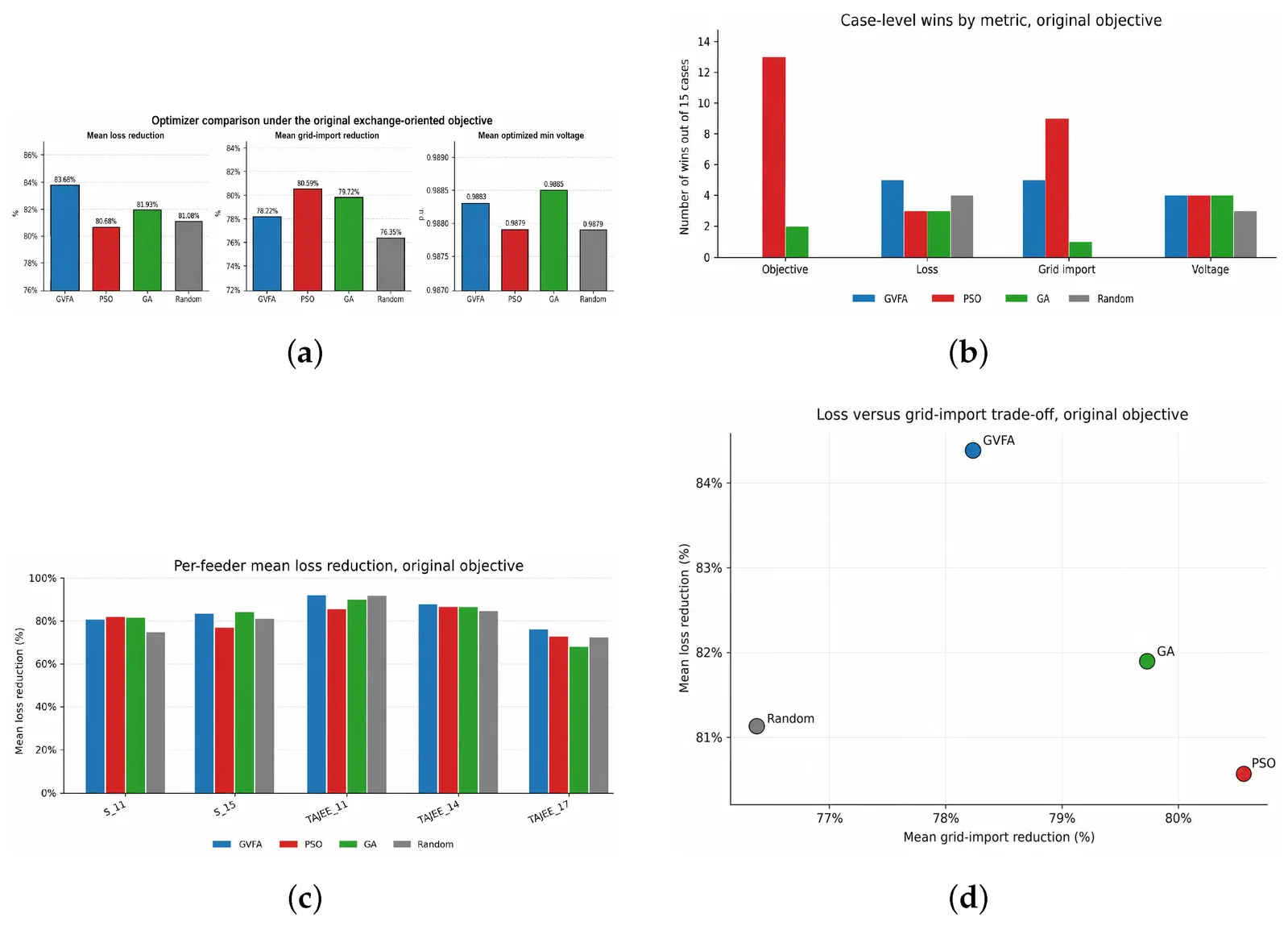
Comparative assessment of GVFA, PSO, GA, and random search under the original exchange-oriented objective. (**a**) Mean optimizer performance in terms of loss reduction, grid-import reduction, and optimized minimum voltage. (**b**) Case-level winning frequency for the overall objective and individual performance metrics across 15 cases. (**c**) Per-feeder mean loss-reduction performance for the practical 11 kV Tajeeyaat/North Baghdad feeder cluster. (**d**) Loss-reduction versus grid-import-reduction trade-off, highlighting the balance between network-loss minimization and upstream-import reduction.

Figure 12a–d summarizes the comparative behavior of the optimizers under the original exchange-oriented objective function. However, because all tested optimizers are stochastic, mean values alone are insufficient to characterize performance stability. Therefore, Table 14 reports descriptive statistics obtained from five practical feeders and three independent random seeds per feeder, resulting in 15 matched feeder–seed cases for each algorithm. All methods used the same population size of 24, iteration limit of 40, decision-variable encoding, objective function, constraints, weather inputs, and seeds 42, 43, and 44.

As shown in Table 14, PSO achieved the lowest mean composite objective and the largest mean grid-import reduction, whereas GVFA achieved the highest mean loss reduction and the lowest mean computational time. GA produced the highest mean minimum voltage, although the difference in optimized voltage among GVFA, PSO, and GA was small. All methods obtained voltage-feasible solutions in the 15 evaluated cases, indicating that the constraint-handling procedure was effective across all optimizers.

The dispersion values in Table 14 are important for interpreting the comparison. The standard deviations overlap across several metrics, especially for the composite objective and grid-import reduction. Therefore, no universal statistical superiority of GVFA over PSO, GA, or other metaheuristic algorithms is claimed. Rather, the comparison demonstrates that GVFA is a competitive and consistently feasible solver under the adopted matched-budget setting. In particular, GVFA provides favurable loss-reduction performance and the lowest mean runtime, while PSO provides stronger performance in the composite objective and grid-import reduction metrics.

This interpretation is consistent with the convergence and trade-off behavior shown in Figure 12. In particular, Figure 12c,d indicate that GVFA provides favorable feeder-loss reduction and a competitive loss/import trade-off, whereas PSO shows stronger performance in reducing residual grid dependence. Therefore, the optimizer comparison should be interpreted as an empirical matched-budget assessment rather than as a theoretical convergence or dominance claim. No theoretical proof of GVFA superiority over PSO, GA, or other population-based methods is claimed in this study. Under the same objective function, decision-variable encoding, constraints, weather inputs, population size, iteration limit, and random-seed protocol, the results indicate that GVFA is a competitive and consistently feasible planning optimizer within the proposed two-stage framework, rather than a universally dominant algorithm.

### 3.6. Limitations and Deployment Requirements

The proposed two-stage GVFA framework is intended as a planning-level decision-support tool for DER allocation, voltage assessment, and preliminary inter-feeder exchange planning. Therefore, the reported results should be interpreted within several modeling and implementation limitations.

First, the load-flow analysis is based on balanced steady-state radial BFS modeling. It evaluates voltage magnitudes, feeder losses, and steady-state feasibility, but does not capture unbalanced three-phase operation, phase-specific loading, neutral-current effects, harmonics, electromagnetic transients, dynamic stability, or fast inverter-control interactions.

Second, the BESS model is simplified and planning-oriented. Storage units are represented by optimized power ratings and prescribed normalized flexibility profiles. Chronological state-of-charge recursion, charging/discharging efficiency, degradation, cycle ageing, converter constraints, replacement cost, and real-time dispatch control are outside the present scope. Thus, BESS-related benefits indicate planning-level network-support potential rather than validated operational battery schedules.

Third, the study does not include protection coordination, short-circuit analysis, anti-islanding assessment, reverse-power protection review, harmonic analysis, phase-imbalance assessment, or thermal validation of lines, transformers, switches, and tie cables. Therefore, the selected tie capacities and exchange corridors should be regarded as planning candidates requiring subsequent utility protection and thermal-rating verification.

Finally, the economic assessment is limited to technical indicators, including losses, voltage profile, grid-import reduction, exchange balance, and ENS. A full CAPEX/OPEX, net-present-value, tariff, ownership, maintenance, land-use, and battery-replacement cost analysis is not included. Consequently, the framework identifies technically promising DER placements and inter-feeder exchange corridors, but it does not represent a final investment or real-time deployment plan. Future work should extend the model toward unbalanced three-phase operation, time-coupled SOC-constrained BESS dispatch, protection-aware and thermal-constrained exchange scheduling, economic planning, and field validation using measured utility data.

## 4. Conclusions

This study developed and evaluated a weather-driven two-stage GVFA framework for planning DER integration and inter-feeder coordination in radial distribution networks. The framework was tested on a severe IEEE-33 benchmark cluster and a practical five-feeder 11 kV Tajeeyaat/North Baghdad system. The results show that coordinated active- and reactive-power resource allocation can reduce aggregate active losses, improve feeder voltage profiles, and identify residual feeder-level surplus and deficit patterns that are not captured by independent-feeder planning alone.

The main conclusions should be interpreted as planning-level findings. In Stage-1, the optimized allocation of PV, WT, BESS, capacitor banks, and auxiliary resources provides candidate DER locations and sizes that improve loss and voltage indicators under the adopted balanced steady-state BFS model. In Stage-2, the exchange results identify candidate feeder-to-feeder corridors, tie capacities, and exporter–importer relationships that indicate where local energy sharing may be technically valuable. These findings support planning decisions related to DER siting, feeder ranking, voltage-support requirements, grid-import reduction, and preliminary inter-feeder exchange screening. The contingency and load-growth analyses provide resilience-related planning indicators rather than direct proof of operational resilience. For the practical Tajeeyaat/North Baghdad cluster, the tested scenarios maintained acceptable voltage margins under the evaluated N-1 and load-growth cases. For the IEEE-33 benchmark cluster, DER-outage and demand-stress cases revealed lower-voltage violations, indicating the need for additional voltage-control measures or reinforcement under more severe operating conditions. These results are useful for comparative robustness screening, but they should not be interpreted as validated service-restoration capability, protection-secure operation, or real-time fault-management performance.

The bidirectional exchange results should also be interpreted within the planning scope of the model. Stage-2 identifies candidate corridors where feeder-to-feeder exchange may reduce residual grid dependence and improve local utilization of DER surplus. However, the reported exchange capacities and signed energy flows are not field-ready switching commands or operational dispatch schedules. Practical bidirectional operation would require time-resolved monitoring, controllable tie switches, bidirectional metering, reverse-power protection review, feeder thermal-limit verification, and utility-approved operating procedures.

Future work should extend the proposed framework from planning-level screening toward operationally validated deployment. A first direction is to replace the prescribed BESS power profile with a time-coupled storage model that includes chronological SOC recursion, charging/discharging efficiency, energy-capacity constraints, degradation, cycle aging, converter limits, and validated dispatch strategies. A second direction is to upgrade the balanced steady-state BFS model to an unbalanced three-phase formulation capable of representing phase-specific loading, neutral-current effects, phase imbalance, harmonics, electromagnetic transients, dynamic stability, and fast inverter-control interactions.

Further development should also integrate protection-aware and thermal-constrained operation. This requires short-circuit analysis, relay-setting coordination, anti-islanding verification, reverse-power protection assessment, fault-current contribution of inverter-based resources, and thermal validation of lines, transformers, switches, and tie cables. In addition, future studies should include a detailed techno-economic layer covering CAPEX/OPEX, net-present value, tariff structures, DER ownership, maintenance cost, battery replacement, land-use cost, and market-participation mechanisms. Finally, field-oriented validation is required using synchronized AMI, SCADA/DMS, DER inverter telemetry, BMS, weather-station or remote-sensing data, capacitor-controller status, tie-switch measurements, and tie-line metering. These extensions would allow the proposed framework to progress from planning-level DER/CAP siting, voltage/loss screening, feeder surplus–deficit analysis, and candidate exchange-corridor identification toward protection-secure, thermally feasible, economically justified, and operationally validated smart-grid deployment.

## Figures and Tables

**Table 1:** Base-case design and modeling assumptions for the IEEE-33 benchmark cluster and the practical Tajeeyaat/North Baghdad feeder cluster.

Item	IEEE-33 Benchmark Cluster	Tajeeyaat/North Baghdad 11-kV Cluster
Base-case purpose	Benchmark validation using five replicated IEEE-33 radial feeders.	Practical validation using five utility-derived 11-kV feeders from the Tajeeyaat/North Baghdad system.
Data source	Embedded IEEE-33 system data generated inside the code.	Utility-derived feeder, node, load, and line records from Baghdad Electricity Distribution Company.
Network size	5 feeders; 33 buses and 32 branches per feeder; 165 buses and 160 branches in total.	5 practical feeders; 191 physical buses in total; branch data reconstructed from the utility workbook.
Voltage level	12.66 kV.	11 kV.
Base active/reactive demand	3715 kW and 2300 kVAr per IEEE-33 feeder before hourly scaling.	Utility-derived feeder-specific P/Q demand.
Load scaling and hourly profiles	IEEE-33 demand is modified using the same hourly/weather-dependent multiplier $\lambda_{m,h}$ used in the study; 24 hourly operating points are evaluated.	Hourly/seasonal planning profiles are derived from available feeder and weather data; 24 hourly operating points are evaluated.
Weather period and source	NASA POWER weather data for Baghdad/North Baghdad, 1 January 2024 to 1 January 2025.	NASA POWER weather data for Baghdad/North Baghdad, 1 January 2024 to 1 January 2025.
Stage-1 resources per feeder	4 PV units, 1 WT, 4 BESS units, 2 capacitor banks, and 1 auxiliary resource.	4 PV units, 1 WT, 4 BESS units, 2 capacitor banks, and 1 auxiliary resource.
Capacity bounds used in optimization	PV: $\leq \;\min(2600,1.05P_{f}^{\mathrm{peak}})$ kW; BESS: $\leq \;\min(2000,0.85P_{f}^{\mathrm{peak}})$ kW; CAP: $\leq \;\min(1400,0.55P_{f}^{\mathrm{peak}})$ kVAr.	Same capacity-bound formulation as the IEEE-33 cluster; practical feeder peak demand is used in the feeder-specific limits.
Candidate buses/nodes	Feasible non-slack IEEE-33 buses; labels are reported as Bxx.	Feasible practical feeder nodes; original utility identifiers are retained, e.g., T11, T17, T14, S15, S11, TR2, and TR3 labels.
Technology rationale	PV and WT represent complementary weather-driven renewable generation; BESSs provide planning-level temporal flexibility; CAP provides reactive-power voltage support; EVCSs, diesel, and biogas represent auxiliary feeder-support scenarios.	Same rationale; PV, WT, BESSs, and CAP are optimized as core planning resources, while EVCSs, diesel, and biogas are treated as feeder-specific auxiliary scenarios.
Auxiliary-resource assumption	EVCS, diesel, or biogas is assigned as a predefined planning scenario, not inferred from measured installed assets.	EVCS, diesel, or biogas is assigned as a predefined planning scenario, not inferred from measured installed assets in the utility data.
Stage-2 exchange variables	Tie-switch status and transfer capacity between candidate feeder pairs.	Tie-switch status and transfer capacity between candidate feeder pairs.
Main modeling scope	Planning-level balanced steady-state BFS assessment with assumed hourly profiles.	Planning-level balanced steady-state BFS assessment with assumed hourly profiles.

**Table 2:** Input variables, present-study data sources, and practical sensing or monitoring requirements.

Input Variable	Source Used in This Study	Practical Sensing or Monitoring System	Expected Time Resolution	Used in Stage
Feeder topology and bus connectivity	Utility records and IEEE-33 data	GIS/asset database, ADMS/DMS topology processor, and switch-status sensors	Updated after network changes; switch status in seconds–minutes	Stage 1 and Stage 2
Branch parameters	Utility line records and benchmark data	Utility asset database, field measurements, and line-parameter records	Static or updated after asset replacement	Stage 1 and Stage 2
Bus and load locations	Utility feeder records and benchmark load data	GIS, transformer/customer database, and AMI mapping	Static or periodically updated	Stage 1
Load demand profile	Base load data with hourly and seasonal scaling	Smart meters, AMI/MDMS, feeder meters, and transformer meters	15 min–1 h	Stage 1 and Stage 2
Weather data	NASA POWER data for Baghdad/North Baghdad	Weather stations, satellite/remote-sensing products, and forecasting platforms	10 min–1 h for operation; daily/monthly for planning	Stage 1
Solar irradiance	NASA POWER-derived profile	Pyranometers, satellite irradiance products, and PV inverter telemetry	10 min–1 h	Stage 1
Wind speed	NASA POWER-derived profile	Anemometers, weather stations, and remote-sensing services	10 min–1 h	Stage 1
PV/WT availability	Weather-dependent resource model	DER inverter telemetry, DERMS, SCADA, and forecasting tools	1–15 min for operation; hourly for planning	Stage 1 and Stage 2
BESS capability	Prescribed planning-level flexibility profile and capacity bounds	BMS, power-conversion-system telemetry, and DERMS	Seconds–15 min	Stage 1 and Stage 2
Capacitor-bank status	Candidate ratings and planning bounds	Capacitor-bank controller, SCADA, and voltage/VAr meters	Seconds–minutes	Stage 1
Auxiliary resources	Planning assumptions for EVCS, diesel, and biogas	EVCS management system, generator controller, and biogas-plant SCADA	1–15 min	Stage 1
Bus voltages and feeder flows	Calculated by BFS load flow	SCADA, feeder meters, voltage sensors, AMI voltage data, and micro-PMU where available	Seconds–15 min	Stage 1 and Stage 2
Tie-switch status	Candidate Stage 2 configuration	SCADA-controlled switches, RTUs, and switch-position sensors	Seconds–minutes	Stage 2
Tie-line loading and exchange capacity	Stage 2 planning limits and calculated flows	Tie-line meters, line sensors, and SCADA/DMS	Seconds–15 min	Stage 2
Surplus/deficit profiles	Computed from optimized feeder generation and demand	DERMS, AMI, feeder metering, and SCADA/DMS energy-balance tools	15 min–1 h	Stage 2
ENS and contingency status	Scenario-based N-1 assessment	OMS, SCADA, fault indicators, and AMI last-gasp signals	Event-based; seconds–minutes	Validation

**Table 3:** Traceability and justification of principal constants and empirical parameters.

Parameter or Constant	Value	Unit	Source/Type	Justification and Interpretation
Voltage lower limit	0.95	p.u.	Planning voltage-security criterion	Defines the minimum acceptable steady-state voltage used for feasibility screening.
Voltage upper limit	1.05	p.u.	Planning voltage-security criterion	Defines the maximum acceptable steady-state voltage used for feasibility screening.
BFS damping coefficient	0.60	–	Numerical implementation parameter	Stabilizes BFS convergence under high DER penetration and reverse-power-flow tendencies.
BFS tolerance	$10^{-7}$	p.u.	Numerical implementation parameter	Ensures repeatable voltage and loss calculations without excessive computational cost.
BFS iteration cap	100	iterations	Numerical safeguard	Prevents non-convergent candidate solutions from consuming unlimited computation time.
PV monthly normalization denominator	5.0	kWh/m^2^/day	Proposed planning normalization	Converts monthly shortwave irradiation into a dimensionless PV availability multiplier around a representative reference value.
PV factor clipping limits	0.35–1.22	–	Proposed planning saturation	Prevents unrealistically low or high monthly PV multipliers while preserving seasonal variation.
WT wind-speed reference	4.0	m/s	Proposed planning normalization	Converts monthly wind speed into a dimensionless wind availability multiplier.
WT monthly factor clipping limits	0.20–1.18	–	Proposed planning saturation	Prevents unrealistically low or excessive seasonal WT availability.
WT output clipping limits	0.05–0.98	–	Proposed planning bound	Avoids negative output and prevents assuming rated WT generation in all hours.
PV reactive-power support ratio	0.18	kVAr/kW	Planning assumption	Represents limited inverter-based reactive-power support without modeling a full inverter capability curve.
WT reactive-power support ratio	0.12	kVAr/kW	Planning assumption	Represents lower reactive-support contribution from WT units at planning level.
BESS reactive-power support ratio	0.25	kVAr/kW	Planning assumption	Represents inverter-coupled reactive support from BESS without detailed converter modeling.
Capacitor minimum availability	0.30	–	Planning approximation	Maintains a minimum reactive-support level in the load-dependent capacitor profile.
Capacitor profile exponent	0.85	–	Planning approximation	Controls the nonlinear increase of capacitor support with loading.
PV capacity cap	2600	kW	Final simulation bound	Limits individual PV unit size to avoid unrealistic oversizing.
BESS capacity cap	2000	kW	Final simulation bound	Limits individual BESS power rating in the planning optimization.
Capacitor-bank cap	1400	kVAr	Final simulation bound	Limits individual capacitor-bank size to avoid excessive reactive injection.
PV peak-load fraction	1.05	p.u. of feeder peak	Technology-sizing bound	Prevents a single PV unit from becoming disproportionately large relative to feeder peak load.
BESS peak-load fraction	0.85	p.u. of feeder peak	Technology-sizing bound	Prevents a single BESS unit from dominating feeder peak demand.
CAP peak-load fraction	0.55	p.u. of feeder peak	Technology-sizing bound	Prevents excessive reactive compensation relative to feeder demand.
Stage 1 loss weight	1.0	–	Proposed objective weight	Establishes active-power loss as the reference technical objective.
Stage 1 voltage-deviation weight	180	–	Proposed objective weight	Gives strong priority to voltage-profile improvement because voltage deviations are numerically small after normalization.
Stage 1 voltage-violation penalty	$10^{6}$	–	Proposed feasibility penalty	Strongly discourages electrically infeasible candidate solutions.
Stage 1 grid-import weight	0.030	–	Proposed objective weight	Rewards reduced upstream dependence while avoiding dominance over voltage feasibility.
Stage 1 siting penalty	45	–	Proposed planning penalty	Penalizes geographically or electrically unsuitable candidate locations.
Stage 2 grid-import weight	0.070	–	Proposed objective weight	Encourages residual grid-import reduction after feeder exchange.
Stage 2 tie-loss weight	1.0	–	Proposed objective weight	Penalizes transfer losses associated with feeder-to-feeder exchange.
Stage 2 unserved-dependency weight	0.12	–	Proposed objective weight	Penalizes the unserved-dependency proxy in exchange planning.
Stage 2 reliability-gain weight	500	–	Proposed objective weight	Rewards configurations that improve the planning-level local supply support proxy.
Stage 2 tie-count weight	25	–	Proposed regularization weight	Avoids unnecessarily dense inter-feeder tie configurations.
Tie-loss factor	0.018	p.u. of exchanged energy	Proposed planning approximation	Represents a 1.8% transfer-loss allowance so that feeder-to-feeder exchange is not treated as lossless.
Switch threshold	0.50	–	Binary decoding rule	Converts a normalized switch gene into an open/closed tie-switch decision.
Minimum tie capacity offset	25	kW	Stage 2 decoding parameter	Avoids nonzero selected ties with negligible exchange capacity.
Cluster capacity fraction	0.35	p.u. of cluster peak	Stage 2 capacity bound	Scales maximum candidate tie capacity with the total cluster demand level.
Minimum cluster capacity limit	50	kW	Stage 2 capacity bound	Provides a lower bound for small candidate exchange capacities.
Disconnected graph penalty	5000	objective units	Proposed feasibility penalty	Penalizes exchange solutions that request transfer through disconnected feeder graphs.
GVFA inertia coefficient	0.55	–	Tuned algorithmic parameter	Preserves part of previous velocity while limiting oscillation.
GVFA learning rate	0.055	–	Tuned algorithmic parameter	Controls leader-attraction strength and balances convergence speed with stability.
Leader-attraction schedule	$1.15-0.35\pi_{t}$	–	Tuned algorithmic schedule	Gradually reduces leader attraction to avoid excessive late-stage oscillation.
Predecessor-following base coefficient	0.22	–	Tuned GVFA coefficient	Represents V-formation following behavior.
Predecessor-following decay factor	$1-0.5\pi_{t}$	–	Tuned GVFA schedule	Reduces predecessor-following influence as the search approaches exploitation.
Wing-offset decay exponent	1.35	–	Tuned exploration schedule	Controls nonlinear decay of asymmetric wing exploration.
Stochastic exploration schedule	$0.35(1-\pi_{t})+0.05$	–	Tuned exploration schedule	Maintains exploration early while preserving a small late-stage random perturbation.
Reinitialization interval	25	iterations	Tuned diversity parameter	Reinitializes the final goose periodically to reduce premature convergence.
Early-stop tolerance	$10^{-5}$	relative fitness change	Tuned stopping parameter	Stops the search only when objective improvement becomes negligible.
Early-stop patience	18	iterations	Tuned stopping parameter	Avoids premature termination due to short-term stochastic fluctuations.
Stage 1 full population/iterations	36/120	geese/iterations	Final simulation setting	Balances feeder-level search diversity with BFS computational burden.
Stage 2 full population/iterations	44/140	geese/iterations	Final simulation setting	Provides broader exploration for tie-state and exchange-capacity decisions.
Random seed	42	–	Reproducibility setting	Ensures repeatable stochastic initialization and fair comparison among runs.

**Table 4:** GVFA algorithmic parameter-sensitivity protocol.

Sensitivity Group	Reference Setting	Tested Variation	Main Purpose	Reported Metrics
Stage 1 population/iterations	$N_{1}=36$, $T_{1}=120$	Screening and full settings	Assess feeder-level search-depth robustness	Final objective, loss reduction, $V_{\min}$, runtime
Stage 2 population/iterations	$N_{2}=44$, $T_{2}=140$	Screening and full settings	Assess exchange-level search-depth robustness	Exchanged energy, residual grid import, tie losses, runtime
Learning rate	α=0.055	Lower and higher values around the reference setting	Test sensitivity to leader-attraction strength	Final objective, convergence iteration, feasibility rate
Reinitialization interval	$T_{\mathrm{ex}}=25$	More frequent and less frequent regeneration	Test diversity-restoration effect	Final objective, stagnation behavior, runtime
Early-stop settings	$\varepsilon_{\mathrm{stop}}=10^{-5}$, p=18	Stricter and looser stopping criteria	Test convergence-stopping robustness	Runtime, final objective, convergence iteration
Tie-loss factor	$\eta_{\mathrm{tie}}=0.018$	Reduced and increased transfer-loss assumptions	Test exchange-planning sensitivity	Exchanged energy, residual import, tie-loss term
Random-seed repeatability	Seed 42 as reference	Multiple seed-controlled repetitions	Quantify empirical stability	Mean objective, standard deviation, $CV_{J}$

**Table 5:** Principal simulation and optimization settings.

Parameter	Symbol	Value	Function
Voltage limits	$V_{\min},V_{\max}$	0.95,1.05p.u.	Feasibility range
BFS damping/tolerance	$\alpha_{\mathrm{BFS}},\varepsilon$	$0.60,10^{-7}$	Stable load-flow convergence
BFS iteration cap	$K_{\max}$	100	Numerical safeguard
Stage-1 population/iterations	$N_{1},T_{1}$	24/80; 36/120 full	Feeder-level search
Stage-2 population/iterations	$N_{2},T_{2}$	32/80; 44/140 full	Exchange-level search
Learning rate	α	0.055	Leader-attraction scaling
Exchange interval	$T_{\mathrm{ex}}$	25	Diversity restoration
Early-stop tolerance/patience	$\varepsilon_{\mathrm{stop}},p$	$10^{-5}$/18	Stagnation control
PV/BESS/CAP units	$N_{\mathrm{PV}},N_{\mathrm{BESS}},N_{\mathrm{CAP}}$	4/4/2	Portfolio cardinality
PV/BESS/CAP caps	–	2600/2000/1400	Per-unit kW/kW/kVAr bounds
Tie-loss factor	$\eta_{\mathrm{tie}}$	0.018	Exchange-loss representation

**Table 6:** Comparative technical summary of the two case studies.

Case Study	Base Loss (kWh-eq/24 h)	Stage-1 Loss (kWh-eq/24 h)	Aggregate Loss Reduction (%)	Base $V_{\min}$ Range (p.u.)	Stage-1 $V_{\min}$ Range (p.u.)	GIR (%)
IEEE-33 benchmark cluster	31,746.4712	6381.1403	79.8997	0.8268–0.8632	0.9465–0.9683	84.4596
Tajeeyaat/North Baghdad	11,258.1571	3367.8481	70.0853	0.9497–0.9898	0.9897–0.9997	94.0270

*Note:* GIR: Grid-import reduction.

**Table 7:** Optimal placement and sizing of PV, WT, BESS, and auxiliary resources in the IEEE-33 benchmark cluster and the Tajeeyaat/North Baghdad 11-kV feeder system.

**(a) IEEE-33 Benchmark Cluster**				
**Fdr.**	**PV Location: Size (kW)**	**WT Location: Size (kW)**	**BESS Location: Size (kW)**	**BIO/DG/EVCS Location: Size (kW)**
1	**Total = 4155.6** B02: 1855.7; B27: 960.3; B06: 491.9; B30: 847.7	**Total = 1364.0** B07: 1364.0	**Total = 2138.8** B33: 395.0; B05: 833.5; B21: 142.7; B24: 767.6	**BIO, Total = 687.3** B14: 687.3
2	**Total = 3363.9** B25: 580.3; B05: 921.4; B03: 474.2; B30: 1388.0	**Total = 2139.6** B04: 2139.6	**Total = 3851.9** B24: 1651.7; B31: 1098.7; B28: 677.9; B32: 423.6	**DG, Total = 1090.5** B08: 1090.5
3	**Total = 5968.2** B10: 904.7; B33: 1640.3; B21: 1389.5; B25: 2033.7	**Total = 305.0** B18: 305.0	**Total = 5369.5** B06: 551.9; B02: 1259.9; B30: 1595.1; B24: 1962.6	**BIO, Total = 1059.3** B05: 1059.3
4	**Total = 3696.0** B22: 909.4; B14: 564.1; B24: 1530.4; B30: 692.1	**Total = 1007.1** B32: 1007.1	**Total = 2447.8** B13: 727.4; B23: 681.4; B27: 140.0; B02: 899.0	**BIO, Total = 1106.6** B06: 1106.6
5	**Total = 3838.5** B33: 767.5; B13: 386.1; B03: 1484.8; B27: 1200.1	**Total = 1310.3** B11:1310.3	**Total = 2780.7** B08: 557.3; B28: 1176.0; B25: 568.7; B23: 478.7	**EVCS, Total = 840.1** B24: 840.1
**(b) Tajeeyaat/North Baghdad 11-kV Feeder System**				
**Fdr.**	**PV Location: Size (kW)**	**WT Location: Size (kW)**	**BESS Location: Size (kW)**	**BIO/DG/EVCS Location: Size (kW)**
T11	**Total = 8659.8** TR2-7: 1673.8; T11-11: 2472.6; T14-39: 2394.9; T11-54: 2118.5	**Total = 2285.0** T11-3: 2285.0	**Total = 6975.6** T11-8: 1610.4; T11-4: 1809.2; T11-18: 1931.3; T11-5: 1624.7	**BIO, Total = 1500.0** T11-61: 1500.0
T17	**Total = 10,388.6** T17-32: 2593.1; T17-11: 2595.7; T17-15: 2599.8; T17-3: 2600.0	**Total = 2496.1** T17-17: 2496.1	**Total = 6951.7** T17-26: 1974.7; T17-27: 1814.1; T17-13: 1741.0; T17-5: 1421.9	**DG, Total = 1800.0** T17-8: 1800.0
T14	**Total = 9687.5** T14-24: 2548.0; B184: 2444.1; T14-27: 2227.0; T14-18: 2468.4	**Total = 2500.0** T14-22: 2500.0	**Total = 6318.2** TR3-8: 691.0; B789: 1932.6; T14-26: 1927.3; T14-23: 1767.3	**BIO, Total = 1483.5** T14-37:1483.5
S15	**Total = 9805.3** S15-48: 2128.9; S15-8: 2600.0; S15-52: 2600.0; S15-19: 2476.4	**Total = 2500.0** S15-31: 2500.0	**Total = 7467.1** S15-7: 1647.3; S15-15: 1959.2; S15-25: 1894.0; S15-38: 1966.6	**EVCS, Total = 27.7** S15-22:27.7
S11	**Total = 10,059.5** S11-44: 2471.9; S11-11: 2600.0; S11-21: 2465.5; S11-3: 2522.1	**Total = 2176.9** S11-33:2176.9	**Total = 5933.0** S11-10: 1936.8; S11-18: 1100.5; S11-28:1713.8; S11-45:1181.9	**EVCS, Total = 92.7** S11-39: 92.7

*Note:* T denotes the Total capacity of each technology within the corresponding feeder. Bxx denotes Bus-xx number for the IEEE-33 benchmark. For the practical case, T11, T17, and T14 denote 11KV_TAJEE_11, 11KV_TAJEE_17, and 11KV_TAJEE_14, respectively; S15 and S11 denote 11KV_SHAMALBAGHDAD_15 and 11KV_SHAMALBAGHDAD_11, respectively. TR2-7 and TR3-8 retain the original transformer-node identifiers. BIO: biogas generation; DG: Diesel Generator; EVCS: Electric-Vehicle Charging Station.

**Table 8:** IEEE-33 feeder-level performance after Stage-1 GVFA optimization.

Feeder	Base Loss (kWh-eq/24 h)	Stage-1 Loss (kWh-eq/24 h)	Loss Reduction (%)	Base $V_{\min}$ (p.u.)	Stage-1 $V_{\min}$ (p.u.)	GIR (%)
IEEE33_FEEDER_1	6299.1402	943.4106	85.0232	0.8454	0.9642	84.5916
IEEE33_FEEDER_2	5615.6217	1597.2092	71.5577	0.8544	0.9606	95.4026
IEEE33_FEEDER_3	7032.7741	1639.5133	76.6875	0.8362	0.9465	86.2174
IEEE33_FEEDER_4	4980.0450	745.5094	85.0301	0.8632	0.9683	91.1360
IEEE33_FEEDER_5	7818.8902	1455.4978	81.3849	0.8268	0.9612	64.9505

*Note:* GIR: Grid-import reduction.

**Table 9:** Tajeeyaat/North Baghdad feeder-level performance after Stage-1 GVFA optimization.

Feeder	Base Loss (kWh-eq/24 h)	Stage-1 Loss (kWh-eq/24 h)	Loss Reduction (%)	Base $V_{\min}$ (p.u.)	Stage-1 $V_{\min}$ (p.u.)	GIR (%)
11KV_TAJEE_11	3171.2399	1188.9384	62.5087	0.9497	0.9926	96.1066
11KV_TAJEE_17	477.3719	513.7915	−7.6292	0.9898	0.9997	99.6889
11KV_TAJEE_14	1804.2922	569.7024	68.4252	0.9704	0.9967	97.3701
11KV_SHAMALBAGHDAD_15	3120.9574	643.4156	79.3840	0.9576	0.9912	89.1748
11KV_SHAMALBAGHDAD_11	2684.2957	452.0002	83.1613	0.9623	0.9897	87.7947

*Note:* GIR: Grid-import reduction.

**Table 10:** Selected Stage-2 tie-switch configurations and transfer capacities.

System	From Feeder	To Feeder	Tie Capacity (kW)	Switch Status
IEEE-33	IEEE33_FEEDER_1	IEEE33_FEEDER_2	6015.4	Closed
IEEE-33	IEEE33_FEEDER_1	IEEE33_FEEDER_3	2845.1	Closed
IEEE-33	IEEE33_FEEDER_2	IEEE33_FEEDER_3	2232.8	Closed
IEEE-33	IEEE33_FEEDER_2	IEEE33_FEEDER_5	4453.1	Closed
IEEE-33	IEEE33_FEEDER_3	IEEE33_FEEDER_5	2220.8	Closed
IEEE-33	IEEE33_FEEDER_4	IEEE33_FEEDER_5	7694.6	Closed
Tajeeyaat	11KV_TAJEE_11	11KV_SHAMALBAGHDAD_11	5817.2	Closed
Tajeeyaat	11KV_TAJEE_17	11KV_SHAMALBAGHDAD_11	6876.2	Closed
Tajeeyaat	11KV_TAJEE_14	11KV_SHAMALBAGHDAD_15	7050.5	Closed
Tajeeyaat	11KV_TAJEE_14	11KV_SHAMALBAGHDAD_11	3943.8	Closed

**Table 11:** Signed annual Stage-2 energy exchange. Positive means export and negative means import.

System	Feeder	Yearly Net Exchange (kWh)
IEEE-33	IEEE33_FEEDER_1	−2,109,970
IEEE-33	IEEE33_FEEDER_2	4,959,966
IEEE-33	IEEE33_FEEDER_3	−52,409
IEEE-33	IEEE33_FEEDER_4	370,373
IEEE-33	IEEE33_FEEDER_5	−3,069,540
Tajeeyaat	11KV_TAJEE_11	773,424
Tajeeyaat	11KV_TAJEE_17	789,480
Tajeeyaat	11KV_TAJEE_14	125,104
Tajeeyaat	11KV_SHAMALBAGHDAD_15	−120,640
Tajeeyaat	11KV_SHAMALBAGHDAD_11	−1,536,983

**Table 12:** Aggregate N-1 and demand-stress outcomes by contingency class. Voltage indicators are evaluated for energized buses, whereas ENS quantifies disconnected or unsupplied load.

System	Contingency Class	Mean 24 h Loss (kWh-eq)	Worst $V_{\min}$ (p.u.)	Maximum ENS (kWh)	Any Voltage Violation
IEEE-33	N-1 line outage	55.1	0.9893	90,558.0	No
IEEE-33	N-1 DER outage	1291.2	0.9249	0.0	Yes
IEEE-33	Load-demand stress	1996.5	0.8872	0.0	Yes
Tajeeyaat	N-1 line outage	42.4	0.9977	126,763.3	No
Tajeeyaat	N-1 DER outage	418.1	0.9744	0.0	No
Tajeeyaat	Load-demand stress	632.1	0.9701	0.0	No

*Note:* For line-outage contingencies, $V_{\min}$ and the voltage-violation flag are evaluated only for buses that remain energized and connected to the slack bus after the outage. ENS represents the load disconnected from the supplied network. Therefore, a line-outage case may show no voltage violation while still producing high ENS. The line-outage loss values should also be interpreted cautiously because lower losses can result from disconnected load rather than improved feeder efficiency.

**Table 13:** Seasonal load-growth sensitivity results for both distribution-system clusters.

System	Season	Load Increase (%)	Total 24 h Loss (kWh-Equivalent)	Minimum Voltage (p.u.)	Grid Import (kWh)	Voltage Violation
IEEE-33	Summer	0.0000	5967.1140	0.9251	99,328.5912	Yes
IEEE-33	Summer	10.0000	7427.3770	0.9083	134,420.5026	Yes
IEEE-33	Summer	20.0000	9534.9032	0.8909	171,480.7080	Yes
IEEE-33	Summer	30.0000	12,330.5717	0.8728	216,412.5810	Yes
IEEE-33	Summer	40.0000	15,860.6690	0.8539	263,502.0345	Yes
IEEE-33	Winter	0.0000	6185.5659	0.9294	173,129.8904	Yes
IEEE-33	Winter	10.0000	7907.5108	0.9143	210,726.5796	Yes
IEEE-33	Winter	20.0000	10,121.4146	0.8987	252,697.5954	Yes
IEEE-33	Winter	30.0000	12,852.4722	0.8825	297,038.7880	Yes
IEEE-33	Winter	40.0000	16,128.5675	0.8657	342,176.5232	Yes
Tajeeyaat	Summer	0.0000	2561.6816	0.9840	37,677.1756	No
Tajeeyaat	Summer	10.0000	2553.5278	0.9815	51,288.5801	No
Tajeeyaat	Summer	20.0000	2842.6444	0.9788	68,633.4751	No
Tajeeyaat	Summer	30.0000	3435.8049	0.9749	89,735.8268	No
Tajeeyaat	Summer	40.0000	4340.0795	0.9698	115,424.8860	No
Tajeeyaat	Winter	0.0000	2159.0050	0.9744	126,569.5530	No
Tajeeyaat	Winter	10.0000	2712.2187	0.9715	153,445.8987	No
Tajeeyaat	Winter	20.0000	3493.3237	0.9685	186,139.4889	No
Tajeeyaat	Winter	30.0000	4506.5577	0.9655	223,930.3991	No
Tajeeyaat	Winter	40.0000	5756.3065	0.9625	263,851.4534	No

**Table 14:** Matched-budget optimizer comparison for the original Stage 1 objective. Values are mean ± sample standard deviation over 15 feeder–seed cases.

Algorithm	Composite Objective ($\times 10^{3}$)	Loss Reduction (%)	Grid-Import Reduction (%)	Minimum Voltage (p.u.)	Feasible Cases	Runtime (s)
GVFA	−64.65±144.38	83.68±6.53	78.22±12.97	0.9883±0.0057	15/15	378.6±48.2
PSO	−139.56±148.88	80.68±6.35	80.59±9.59	0.9879±0.0058	15/15	396.8±74.1
GA	−112.29±150.05	81.93±7.72	79.72±10.43	0.9885±0.0053	15/15	418.6±107.2
Random search	−25.86±133.80	81.08±7.44	76.35±13.34	0.9879±0.0057	15/15	406.9±78.5

*Note:* Lower composite-objective values are better. Bold values identify the best mean result for each metric. The statistics combine five feeders and three independent seeds per feeder.

## Data Availability

The data supporting the findings of this study are available from the corresponding author upon reasonable request.
